# Quantifying Collision Frequency and Intensity in Rugby Union and Rugby Sevens: A Systematic Review

**DOI:** 10.1186/s40798-021-00398-4

**Published:** 2022-01-20

**Authors:** Lara Paul, Mitchell Naughton, Ben Jones, Demi Davidow, Amir Patel, Mike Lambert, Sharief Hendricks

**Affiliations:** 1 Division of Physiological Sciences, Department of Human Biology, Faculty of Health Sciences, University of Cape Town, Cape Town, South Africa; 2 School of Health and Behavioural Sciences, University of the Sunshine Coast, Sippy Downs, QLD Australia; 3 Centre for Human Factors and Sociotechnical Systems, University of the Sunshine Coast, Sippy Downs, QLD Australia; 4 School of Science and Technology, University of New England, Armidale, NSW Australia; 5 Carnegie Applied Rugby Research (CARR) Centre, Carnegie School of Sport, Leeds Beckett University, Leeds, UK; 6 Leeds Rhinos Rugby League Club, Leeds, UK; 7 England Performance Unit, The Rugby Football League, Leeds, UK; 8 Health Through Physical Activity, Lifestyle and Sport Research Centre (HPALS), Department of Human Biology, Faculty of Health Sciences, University of Cape Town, Cape Town, South Africa; 9 Department of Electrical Engineering, African Robotics unit, University of Cape Town, Western Cape, South Africa

**Keywords:** Rugby, Microtechnology, Video-based analysis, Collisions, Training, Injury prevention

## Abstract

**Background:**

Collisions in rugby union and sevens have a high injury incidence and burden, and are also associated with player and team performance. Understanding the frequency and intensity of these collisions is therefore important for coaches and practitioners to adequately prepare players for competition. The aim of this review is to synthesise the current literature to provide a summary of the collision frequencies and intensities for rugby union and rugby sevens based on video-based analysis and microtechnology.

**Methods:**

A systematic search using key words was done on four different databases from 1 January 1990 to 1 September 2021 (PubMed, Scopus, SPORTDiscus and Web of Science).

**Results:**

Seventy-three studies were included in the final review, with fifty-eight studies focusing on rugby union, while fifteen studies explored rugby sevens. Of the included studies, four focused on training—three in rugby union and one in sevens, two focused on both training and match-play in rugby union and one in rugby sevens, while the remaining sixty-six studies explored collisions from match-play. The studies included, provincial, national, international, professional, experienced, novice and collegiate players. Most of the studies used video-based analysis (*n* = 37) to quantify collisions. In rugby union, on average a total of 22.0 (19.0–25.0) scrums, 116.2 (62.7–169.7) rucks, and 156.1 (121.2–191.0) tackles occur per match. In sevens, on average 1.8 (1.7–2.0) scrums, 4.8 (0–11.8) rucks and 14.1 (0–32.8) tackles occur per match.

**Conclusions:**

This review showed more studies quantified collisions in matches compared to training. To ensure athletes are adequately prepared for match collision loads, training should be prescribed to meet the match demands. Per minute, rugby sevens players perform more tackles and ball carries into contact than rugby union players and forwards experienced more impacts and tackles than backs. Forwards also perform more very heavy impacts and severe impacts than backs in rugby union. To improve the relationship between matches and training, integrating both video-based analysis and microtechnology is recommended. The frequency and intensity of collisions in training and matches may lead to adaptations for a “collision-fit” player and lend itself to general training principles such as periodisation for optimum collision adaptation.

*Trial Registration* PROSPERO registration number: CRD42020191112.

**Supplementary Information:**

The online version contains supplementary material available at 10.1186/s40798-021-00398-4.

## Key Points

- In this systematic review of collision frequency and intensity in rugby union and rugby sevens, only four studies quantified collision frequencies and/or intensities in training, three focused on both training and match-play, while 66 studies quantified frequencies and/or intensities of collisions in matches. Further investigation is needed to improve and understand the relationship between training and matches.
- Per minute, rugby sevens players perform more tackles and ball carries into contact than rugby union players and forwards experienced more impacts and tackles than backs. Forwards also perform more very heavy impacts and severe impacts than backs in rugby union.
- Integrating video-based analysis and microtechnology is recommended, and the metrics and grouping variables between training and matches should be consistent.
- The frequency and intensity of collisions in training and matches may lead to adaptations for a “collision-fit” player and lend itself to general training principles such as periodisation for optimum collision adaptation.

## Background

Rugby union and rugby sevens (henceforth called sevens) are invasion team sports that are characterised by frequent high speed running and physical collisions [1, 2]. Although the two rugby codes differ in match duration (sevens = 14 min; rugby union = 80 min) and player numbers (sevens = 7 players; rugby union = 15 players) [3–6], the type of collisions are similar (i.e., tackles, scrums, rucks and mauls) [6]. Winning these collisions is associated with overall team success and player performance [7–9]. For example, Ortega et al. (2009) identified that winning teams complete more tackles than losing teams [7]. These collisions are also physically and technically demanding for players with an associated high injury incidence and burden (injury incidence rate X mean severity) [10–13]. For instance, in senior professional male rugby union players, 29.0 injuries per 1000 player hours occur when being tackled, 19.0 injuries per 1000 player hours occur when tackling and 17.0 injuries per 1000 player hours occur in the ruck/maul [14]. In sevens, 40.4 injuries per 1000 player hours occur when tackling, with 1.2 injuries per 1000 player hours occurring in the mauls and scrums [15].

Given the high injury incidence and burden, and the positive performance outcomes associated with winning collisions in rugby union and sevens, it is important for coaches and practitioners to adequately prepare players for competition. To do this, they need to know the frequency and intensity of these collisions in both training and matches [16]. In matches and training, the frequency and intensity of collisions have been quantified primarily using two methods: video-based analysis and microtechnology. Quantifying the frequency and intensity of collisions using video-based analysis requires the systematic observation and interpretation of video from matches and/or training [17, 18]. Analysing collisions can occur while the matches or training session(s) are underway, although most detailed analyses occur post-match [17]. Previously, video-based analysis was the main method used to quantify collisions in both rugby cohorts [17]. Quantifying collisions in this manner however, is based on human observation, and as such, it is labour intensive and requires reliability checking to reduce bias and subjectivity [16]. For these reasons, a shift to automated methods of collecting collision data through the use of microtechnology has occurred.

In sport, microtechnology typically incorporates global positioning systems (GPS) and micro-electrical mechanical systems (MEMs) that capture the external physical demands of competition and training [19]. Commercially available microtechnology devices for team sports are designed to be unobstructive, so players can wear them during competition and training. One of the first studies using microtechnology to determine physical demands in rugby union was published in 2009 [20], and since then, research using these devices has grown [19]. Initially, GPS was only used to provide information on distance and speed [21, 22]. Since then, MEMs have been built into GPS devices which now house triaxial accelerometers, gyroscopes and magnetometers [22]. Triaxial accelerometers measure acceleration in three different axes (anterior–posterior, medial–lateral and vertical) [16, 22], and the sum of the acceleration in these three axes provides a vector magnitude (g force). This vector magnitude can be used to quantify the intensity of the collision [19, 22]. Each manufacturer has a different algorithm that is used to quantify collisions [23]. As a consequence, validating collision metrics for these devices has been challenging [23]. Although quantifying collisions using microtechnology may be more time efficient than video-based methods, the validity and reliability of microtechnology in rugby union and sevens requires further investigation [16, 24] due to the ambiguity in the current results [25].

To benefit coaches and practitioners, and aid injury prevention and injury management strategies, a synthesis of the frequency and intensity of collisions in rugby union and sevens to date, both in training and matches, is required. For example, a coach who understands the positional match tackle frequencies and intensities can optimise tackle training sessions to meet those position specific match demands. Since one of the roles of coaches and practitioners is to ensure positive adaptations to training and reduce maladaptation, understanding the frequency and intensity of collisions may also aid optimising recovery between training and matches. Therefore, the aim of this systematic review to synthesise the collision frequencies and intensities for rugby union and rugby sevens based on video-based analysis and microtechnology.

## Methods

### Search Strategy

The search strategy was based on a similar systematic review in rugby league [16]. The current systematic review was carried out in accordance with the PRISMA guidelines [28]. The search was conducted from 1 January 1990 to 1 September 2021 on four different electronic databases (PubMed, Scopus, SPORTDiscus and Web of Science). The search used the following combined key terms for collisions (‘tackl*’ OR ‘collision’ OR ‘impact*’) AND (‘dose’ OR ‘frequency’ OR ‘intensity’ OR ‘demands’) AND rugby union (‘rugby’ OR ‘rugby union’ OR ‘rugby sevens’). For example, in PubMed the search was (((tackl* OR collision OR impact* OR collisions)) AND (dose OR frequency OR intensity OR demands)) AND (rugby OR rugby union OR rugby sevens). The reference list of the final full-text articles (*n* = 73) was also examined.

### Selection of Studies

**Fig. 1:**
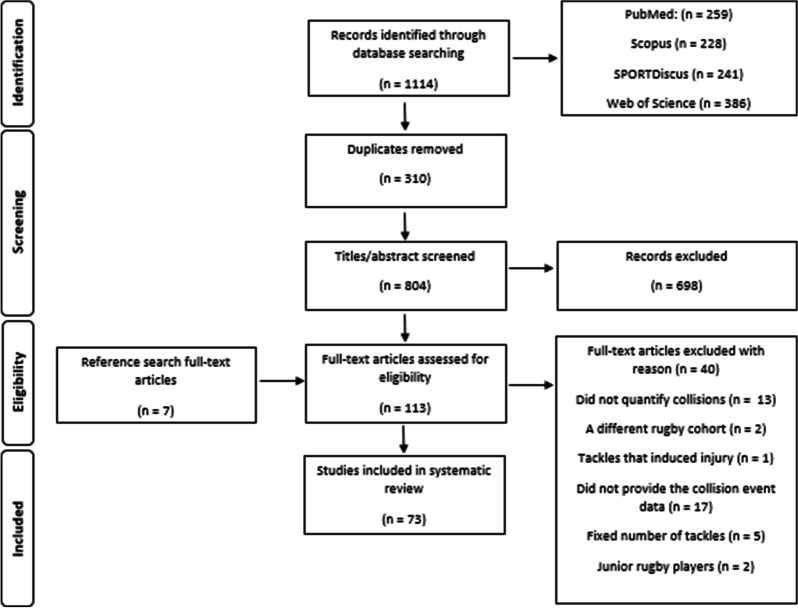
Literature selection process for the systematic review

After consolidating the studies from the different electronic databases, LP removed the duplicates and screened the titles and abstracts (Fig. 1) for eligibility before retrieving the full text [28]. The review was registered with PROSPERO (registration number: CRD42020191112). The full text articles were further screened for eligibility by LP and MN. Any discrepancies in the screening process were discussed until agreed upon. A third researcher was available if consensus on the inclusion of an article could not be reached; however this was not required. The inclusion criteria were (i) any publication that quantified collisions in terms of frequency or intensity in rugby union and/or sevens (ii) study participants within each study had to be over 18 years of age. When collisions were based on ‘impact metrics’, only impacts > 8 g were included in the data to eliminate possible confusion with running demands (i.e., high intensity accelerations or decelerations) unless stated otherwise [25]. Publications from conferences and annual meetings were excluded. Only peer-reviewed publications were included. Any publication that could not be translated into English was excluded. Authors were contacted for detailed information if necessary. The final full-text articles went through the data extraction process.

Collisions were broadly defined as any physical contact made with another player (teammate or opposition), which resulted in an alteration to the player’s momentum. This included collisions such as the tackle (tackling and being tackled), scrums, rucks and mauls [26, 27]. For this review the studies did not need to have a definition to be included.

### Data Extraction

Data relating to participant characteristics (i.e., number, age, height, weight, level of competition, sex, cohort), context (i.e., match play or training), method used to quantify the collisions (i.e., video or microtechnology), the model and specifics of the device (i.e., GPS device rate, inertial sensors, number of files, software), video-based analysis characteristics (i.e., camera system, number of cameras, location of the devices and software), and collision characteristics were extracted from the final 73 full-text articles. Collision characteristics included type of collision, number of matches or training sessions, year of competition, absolute frequency (number), collisions in relation to playing time (number of collisions per minute) and the intensity of each collision. Collision intensity was commonly classified as *very heavy* (8–10 g), *severe* (> 10 g) or *another range* that was specific to the device based on the nature of the collision [29].

### Assessment of Methodological Quality

The quality of the included studies was assessed using the checklist of Downs and Black’s assessment of methodological quality [30]. Questions 5, 8, 9, 13–15, 19, 21–28 were inapplicable due to the nature of the studies. The assessment was done by LP and MN (Additional file 1: Table S1). No studies were eliminated based on the methodological quality.

### Data Analysis

All data were reported in the tables as mean ± standard deviation (SD) unless stated otherwise. Where possible, a meta-analysis (OpenMeta[Analyst]) was completed to produce a pooled mean and 95% confidence intervals (CI). An analysis was only conducted if there were at least two studies with mean and standard deviations. The DerSimonian-Laird continuous random-effects analysis method was used for the meta-analysis, with *I*-squared (*I*^2) used to assess the heterogeneity of the data. *I*^2 of 0–40% was considered low heterogeneity, 40–75%: moderate heterogeneity and > 70% was considered high heterogeneity [16]. The forest plots (mean and 95% CI) presented the results of the meta-analysis.

## Results

### Identification of Studies

The literature search captured 1114 papers (Fig. 1). After the screening process, 73 publications were included in the final review [3, 5, 8, 20, 23–25, 29, 31–95].

### Study Characteristics

In total, 6212 participants were recorded throughout the seventy-three studies (Table 1). Fifteen studies explored sevens (21%) [3, 5, 35–38, 47, 51, 60, 62, 67, 70–72, 78] while fifty-eight studies investigated rugby union (79%) [8, 20, 23–25, 29, 31–34, 39–46, 48–50, 52–59, 61, 63–66, 68, 69, 73–77, 79–95]. Four studies (5%) focused on training (three in rugby union [32, 80, 90] and one in sevens [47]), while two studies investigated training and matches in rugby union (4%) [34, 42] and one in sevens (1%) [51]. The other sixty-six studies (90%) focused on match-play only [3, 5, 8, 20, 23–25, 29, 31, 33, 35–41, 43–46, 48–50, 52–79, 81–89, 91–95]. The studies included, provincial, national, international, professional, experienced, novice and collegiate players. Studies were recorded from the Super Rugby competition [29, 31, 41, 43, 49, 50, 55, 59, 73, 75], Six Nations Championship [8, 33, 88], English Premiership [45, 46, 48, 68], World Rugby Sevens World Series [3, 51, 72], Bledisloe Cup [63], Pro14 [23], and the Rugby World Cup [92, 93].

**Table 1:** Characteristics of studies that were included

Study: author (year)	Number of participants	Male or female	Participant competition level	Age (years): mean ± SD	Height (cm): mean ± SD	Body mass (kg): mean ± SD	Method of data capture	Cohort	Match-play/training or both
Austin et al. (2011) [31]	20	NR	Super 14	Front row forwards: 23 ± 2	Front row forwards: 183 ± 2	Front row forwards: 144 ± 4	Video	Rugby union	Match-play
				Back row forwards: 26 ± 3	Back row forwards: 183 ± 4	Back row forwards: 103 ± 9			
				Inside backs: 22 ± 1	Inside backs: 179 ± 6	Inside backs: 87 ± 3			
				Outside backs: 24 ± 3	Outside backs: 182 ± 4	Outside backs: 100 ± 12			
Bradley et al. (2015) [32]	44 (24 forwards, 20 backs)	NR	Elite	21–34	Forwards: 189 ± 0.6	Forwards: 110.1 ± 6.1	Microtechnology	Rugby union	Training
					Backs: 183 ± 0.5	Backs: 92.1 ± 7			
Bradley et al. (2017) [33]	NR	NR	Six Nation Championship	NR	NR	NR	Video	Rugby union	Match-play
Campbell et al. (2017) [34]	32	Male	Premier Grade Club	24 ± 4	177 ± 10	88 ± 20	Microtechnology and video	Rugby union	Both
Clarke et al. (2015) [35]	12 National	Female	State and National	National: 22.3 ± 2.5	National: 167 ± 0.4	National: 65.8 ± 4.6	Microtechnology	Sevens	Match-play
	10 State			Sate: 24.4 ± 4.3	State: 167 ± 0.3	State: 66.1 ± 7.9			
Clarke et al. (2015) [36]	12 National	Female	State and National	National: 22.3 ± 2.5	National: 167 ± 0.4	National: 65.8 ± 4.6	Microtechnology	Sevens	Match-play
	10 State			Sate: 24.4 ± 4.3	State: 167 ± 0.3	State: 66.1 ± 7.9			
Clarke et al. (2016) [37]	12 males	Male and female	International	Male: 24.1 ± 3.2	Male: 184 ± 0.8	Male: 92 ± 6.9	Microtechnology and video	Sevens	Match-play
	12 females			Female: 22.8 ± 3.6	Female: 169 ± 0.2	Female: 68.6 ± 4.4			
Clarke et al. (2017) [38]	64	Male and female	Domestic and International	NR	Senior Male: 181 ± 0.5	Senior Male: 88.5 ± 10.2	Microtechnology	Sevens	Match-play
					Elite Male: 184 ± 0.7	Elite Male: 92 ± 6.9			
					Senior Female: 170 ± 0.7	Senior Female: 70.4 ± 9.3			
					Elite Female: 169 ± 0.2	Elite Female: 68.6 ± 4.4			
Coughlan et al. (2011) [39]	2 (one forward, one back)	NR	International	30	Forward: 198	Forward: 111.8	Microtechnology and video	Rugby union	Match-play
					Back: 181	Back: 94.9			
Cunniffe et al. (2009) [20]	3	NR	Elite	25 ± 3.6	193.3 ± 9.7	104.6 ± 10.4	Microtechnology	Rugby union	Match-play
Deutsch et al. (1998) [40]	24	Male	Under 19	18.4 ± 0.5	185 ± 7	8.7 ± 9.9	Video	Rugby union	Match-play
Deutsch et al. (2007) [41]	Forwards: 16	NR	Super 12	NR	NR	NR	Video	Rugby union	Match-play
	Backs: 13								
Dubois et al. (2020) [42]	14 Forwards: 6 Backs: 8	NR	Professional	26.9 ± 1.9	185 ± 7.9	97.6 ± 13.2	Microtechnology	Rugby union	Both
Duthie et al. (2005) [43]	47	NR	Super 12	NR	NR	NR	Video	Rugby union	Match-play
Eaton et al. (2006) [44]	35	NR	Professional	20–34 years	NR	NR	Video	Rugby union	Match-play
Fuller et al. (2007) [45]	645	NR	English Premiership	NR	NR	NR	Video	Rugby union	Match-play
Fuller et al. (2008) [46]	645	NR	English Premiership	NR	NR	NR	Video	Rugby union	Match-play
Gibson et al. (2015) [47]	12	Male	International	27.8 ± 3.9	177.8 ± 5.9	81 ± 8.3	Microtechnology	Sevens	Training
Grainger et al. (2018) [48]	38	NR	English Premiership	26.4 ± 4.7	182.3 ± 30.2	100 ± 11	Microtechnology	Rugby union	Match-play
Hendricks et al. (2013) [49]	NR	NR	Super 14	NR	NR	NR	Video	Rugby union	Match-play
Hendricks et al. (2014) [50]	NR	NR	Super 14	NR	NR	NR	Video	Rugby union	Match-play
Hendricks et al. (2018) [8]	NR	NR	Six Nations and Championship	NR	NR	NR	Video	Rugby union	Match-play
Hendricks et al. (2019) [3]	NR	NR	Rugby Sevens World Series	NR	NR	NR	Video	Sevens	Match-play
Higham et al. (2014) [5]	196	Male	International	NR	NR	NR	Video	Sevens	Match-play
Higham et al. (2016) [51]	42	Male	International (World Rugby Sevens World Series and Federation of Oceania Rugby Unions Oceania Sevens Championship)	Forwards: 21.6 ± 2.4	Forwards: 185 ± 0.5	Forwards: 95.8 ± 6.7	Microtechnology	Sevens	Both
				Backs: 21 ± 2.2	Backs: 181 ± 0.6	Backs: 86.2 ± 5.6			
Jones et al. (2014) [52]	28	Male	European Cup	Forwards: 26.7 ± 2.8	NR	Forwards: 111.6 ± 5.7	Microtechnology and video	Rugby union	Match-play
				Backs: 23.4 ± 2.6		Backs: 94.2 ± 7.9			
Jones et al. (2015) [53]	33	NR	Professional	25 ± 4	NR	104 ± 10.6	Microtechnology	Rugby union	Match-play
Lacome et al. (2016) [54]	375	Male	International	NR	NR	NR	Video	Rugby union	Match-play
Lindsay et al. (2015) [55]	37	NR	Super 15	Front row: 26.6 ± 3.7	Front row: 186 ± 0.4	Front row: 112.1 ± 5.1	Video	Rugby union	Match-play
				Locks: 23.7 ± 2.1	Locks: 201 ± 0.5	Locks: 112.3 ± 3.5			
				Loose forwards: 27 ± 4.4	Loose forwards: 188 ± 0.4	Loose forwards: 106.5 ± 2.3			
				Inside backs: 27.5 ± 2.7	Inside backs: 181 ± 0.2	Inside backs: 92.9 ± 3			
				Outside backs: 25.8 ± 1.3	Outside backs: 189 ± 0.5	Outside backs: 106.3 ± 13.7			
Lindsay et al. (2017) [56]	37	NR	Professional	26 ± 3.5	186 ± 0.7	104.5 ± 9.3	Microtechnology and video	Rugby union	Match-play
MacLeod et al. (2018) [25]	37	Male	Professional	27.9 ± 3.6	185.4 ± 7	103.1 ± 12.1	Microtechnology and video	Rugby union	Match-play
McIntosh et al. (2010) [57]	NR	NR	Club Level	NR	NR	NR	Video	Rugby union	Match-play
McLaren et al. (2015) [58]	28 Forwards: 15 Backs: 13	Male	Professional	27 ± 4	187 ± 8	101 ± 14	Microtechnology	Rugby union	Match-play
McLellan et al. (2013) [29]	5	Male	Super 15	Forwards: 23 ± 0.2	Forwards: 193 ± 6.1	Forwards: 116 ± 1.4	Microtechnology	Rugby union	Match-play
				Backs: 22.3 ± 1.5	Backs: 187 ± 1.2	Backs: 93.7 ± 1.5			
Owen et al. (2015) [59]	33	Male	Super 14	25.2 ± 3.5	179.8 ± 33	101.2 ± 13.2	Microtechnology	Rugby union	Match-play
Peeters et al. (2019) [60]	15	Male	Elite	25.8 ± 3.6	182 ± 1	88.9 ± 13.5	Video	Sevens	Match-play
Pollard et al. (2018) [61]	22	Male	International	27 ± 2.9	187 ± 7	106.1 ± 14.1	Microtechnology	Rugby union	Match-play
Portillo et al. (2016) [62]	16	Female	National	23 ± 2	166 ± 7	66 ± 7	Microtechnology	Sevens	Match-play
Quarrie et al. (2007) [63]	NR	NR	Bledisloe Cup	NR	NR	NR	Video	Rugby union	Match-play
Quarrie et al. (2008) [64]	NR	NR	Professional	NR	NR	NR	Video	Rugby union	Match-play
Quarrie et al. (2012) [65]	763	NR	National	NR	NR	NR	Video	Rugby union	Match-play
Reardon et al. (2017) [24]	36	NR	Elite	Forwards: 27.2 ± 3.9	Forwards: 188 ± 0.8	Forwards: 111.6 ± 9	Microtechnology and video	Rugby union	Match-play
				Backs 26.4 ± 5.1	Backs: 181 ± 0.4	Backs: 92 ± 7.4			
Reardon et al. (2017) [66]	39	NR	Elite	27.2 ± 3.9	185 ± 4.3	99.2 ± 24.4	Microtechnology and video	Rugby union	Match-play
Reyneke et al. (2018) [67]	15	Female	International	24.3 ± 3.9	168 ± 7.1	67.5 ± 6.3	Microtechnology and video	Sevens	Match-play
Roberts et al. (2008) [68]	29 Forwards: 14 Backs: 15	NR	English Premiership	NR	NR	NR	Video	Rugby union	Match-play
Roberts et al. (2014) [69]	NR	Male	English community level (3–9)	NR	NR	NR	Video	Rugby union	Match-play
Ross et al. (2015) [70]	84	NR	International and Provincial	NR	NR	NR	Video	Sevens	Match-play
Ross et al. (2015) [71]	27	Male	International	Forwards: 24.4 ± 3.3	Forwards: 188 ± 4.8	Forwards: 95.4 ± 6.3	Video	Sevens	Match-play
				Backs: 23.3 ± 2.9	Backs: 183 ± 4.2	Backs: 89.7 ± 5.9			
Ross et al. (2016) [72]	NR	NR	IRB Sevens World Series	NR	NR	NR	Video	Sevens	Match-play
Schoeman et al. (2015) [73]	15	NR	Super Rugby	NR	NR	NR	Video	Rugby union	Match-play
Smart et al. (2008) [74]	23	Male	New Zealand National Provincial Championship	25 ± 3	184 ± 9	99.2 ± 10.1	Video	Rugby union	Match-play
Smart et al. (2014) [75]	510	NR	Super 14	NR	NR	NR	Video	Rugby union	Match-play
Suarez-Arrones et al. (2012) [76]	9	NR	National	25.9 ± 4	181.5 ± 6.2	90.8 ± 4.8	Microtechnology	Rugby union	Match-play
Suarez-Arrones et al. (2013) [77]	8	Woman	National	Forwards: 26.6 ± 1.9	Forwards: 173.8 ± 5.9	Forwards: 76.8 ± 10.4	Microtechnology	Rugby union	Match-play
				Backs: 27 ± 2.6	Backs: 170 ± 2.3	Backs: 68 ± 3.6			
Suarez-Arrones et al. (2014) [78]	10	Male	National	27.4 ± 1.6	180.4 ± 7.8	87.9 ± 11	Microtechnology and video	Sevens	Match-play
Takarada (2003) [79]	14	NR	Elite	23–30	179.8 ± 1	87.4 ± 2.2	Video		Match-play
Takeda et al. (2014) [80]	20	Male	Collegiate	20 ± 0.6	174 ± 0.5	85.4 ± 2	Microtechnology	Rugby union	Training
Tee et al. (2015) [81]	19	NR	Professional	26 ± 2	186 ± 0.7	101.5 ± 12.2	Microtechnology	Rugby union	Match-play
Tee et al. (2017) [82]	19	NR	Professional	26 ± 2	186 ± 0.7	101.5 ± 12.2	Microtechnology	Rugby union	Match-play
Tee et al. (2020) [83]	19	NR	Professional	26 ± 2	186 ± 0.7	101.5 ± 12.2	Microtechnology	Rugby union	Match-play
Tierney et al. (2020) [23]	44		Guinness PRO14	25.7 ± 3.9	187.0 ± 7.6	102.6 ± 12.0	Microtechnology and video	Rugby union	Match-play
Tierney et al. (2021) [84]	118	Male	Elite	24.7 ± 4.1	186.5 ± 7.0	101.6 ± 12.2	Micotechnology	Rugby union	Match-play
Tucker et al. (2017) [85]	NR	NR	International and National	NR	NR	NR	Video	Rugby union	Match-play
Van Rooyen et al. (2008) [86]	10	NR	Professional	23 ± 3	184 ± 8	99 ± 15	Video	Rugby union	Match-play
Van Rooyen et al. (2012) [87]	NR	NR	International	NR	NR	NR	Video	Rugby union	Match-play
Van Rooyen et al. (2014) [88]	NR	NR	Six Nations	NR	NR	NR	Video	Rugby union	Match-play
Vaz et al. (2010) [89]	NR	NR	International Rugby Board competitions and Super 12	NR	NR	NR	Video	Rugby union	Match-play
Vaz et al. (2012) [90]	40	NR	Experienced and novice	21.6 ± 3.6	177.7 ± 7.4	81.2 ± 10.2	Microtechnology and video	Rugby union	Training
Venter et al. (2011) [91]	17	Male	Provincial	18.5 ± 0.5	183 ± 6	89.8 ± 10.8	Microtechnology	Rugby union	Match-play
Villarejo et al. (2013) [92]	626	NR	Rugby World Cup	NR	NR	NR	Video	Rugby union	Match-play
Villarejo et al. (2015) [93]	736	Male	Rugby World Cup	NR	NR	NR	Video	Rugby union	Match-play
Virr et al. (2014) [94]	38	Female	Premier division club level	24.1 ± 4	168.7 ± 6.5	73.4 ± 10.9	Video	Rugby union	Match-play
Yamamoto et al. (2020) [95]	298	Male	Elite	Forwards: 27.9 ± 3.0	Forwards: 183.1 ± 6.3	Forwards: 100.3 ± 7.2	Microtechnology	Rugby union	Match-play
				Backs: 27.7 ± 2.7	Backs: 173.9 ± 7.8	Backs: 84.2 ± 11.8			

*NR* not reported

Twenty-four studies used microtechnology as a method to record collision demands (33%) [20, 29, 32, 35, 36, 38, 42, 47, 48, 51, 53, 58, 59, 61, 62, 76, 77, 80–84, 91, 95]

and thirty-seven studies used video-based analysis (51%) [3, 5, 8, 31, 33, 40, 41, 43–46, 49, 50, 54, 55, 57, 60, 63–65, 68–75, 79, 85–89, 92–94] (Table 1). Twelve studies used both microtechnology and video-based analysis to capture collision demands (16%) [23–25, 34, 37, 39, 52, 56, 66, 67, 78, 90]. Seven studies (21%) used the GPSports’ SPI Pro device [29, 39, 81–83, 90, 91] and GPSports’ SPI HPU [34–38, 42, 59], 18% used Catapult Minimax S4 [32, 47, 52, 53, 56, 58] and 12% used the StatSports GPS technology [25, 48, 61, 84]. Specifics of both the microtechnology device and software used are provided in Additional file 1: Table S2. Similarly, camera specifics and the video-based analysis system used can be found in Additional file 1: Table S3.

### Microtechnology

#### Rugby Union Match-Play

Ten studies recorded collision frequency using microtechnology in match-play (14%) [20, 23–25, 39, 52, 53, 58, 84, 91] (Table 2). Two studies in rugby union recorded collisions per match [23, 39], while two recorded per position [24, 25]. One study recorded the impacts per min (0.7 ± 0.4 impacts per min) [58]. Macleod et al. (2018) recorded the frequency of collisions per minute per position [25]. Tackles per match [39, 52] and impacts per match [52] for forwards and backs were recorded [20, 39]. Three studies recorded load per collision [25, 39, 84].

**Table 2:** Characteristics of collision frequency detected by microtechnology in rugby union and rugby sevens

Study: author (year)	Number of matches/training sessions	Type of collisions	Frequency definition	Frequency of collisions: mean ± SD		Relative frequency of collisions: mean ± SD (no. per min)		Load (AU)	
*Rugby union*									
Bradley et al. (2015) [32]	Training sessions	Contact number	Weekly	Forwards: 80 ± 25		NR		NR	
				Backs: 50 ± 22					
Coughlan et al. (2011) [39]	1 match	Collisions	Number	Total: 1411		NR		NR	
				Forwards: 838					
				Backs: 573					
		Tackles	Total	Forwards: 10					
				Backs: 12					
		Average Body Load tackle against						Forwards: 8.4 G	
								Backs: 7.8 G	
Cunniffe et al. (2009) [20]	1 match	Impacts	Total	Forwards: 798		NR		NR	
				Backs: 1274					
Jones et al. (2014) [52]	4 matches			Forwards:	Backs:	NR		NR	
		Tackles	Per match	5 ± 3	4 ± 3				
		Contacts hit	Per match	15 ± 6	6 ± 4				
		Impacts	Total	25 ± 9	15 ± 7				
		Scrum	Per match	13 ± 5	0				
		Contacts	Total	31 ± 14	16 ± 7				
Jones et al. (2015) [53]	71 matches	Contacts	Per match	First half: 12.3 ± 9.5		NR		NR	
				Second half: 12.6 ± 9.8					
			0–10 min	2.9 ± 2.5					
			10–20 min	3.1 ± 3					
			20–30 min	4.1 ± 4.6					
			30–40 min	3.7 ± 5					
			40–50 min	4 ± 3.8					
			50–60 min	2.5 ± 2.2					
			60–70 min	2.3 ± 2.1					
			70–80 min	2.5 ± 2.4					
MacLeod et al. (2018) [25]	11 matches	Collisions	Number per game	Forwards:	Backs:	Forwards:	Backs:		
				Prop: 31 ± 6	Half back: 16 ± 5	Prop: 0.4 ± 0.1	Half back: 0.2 ± 0.1		
				Hooker: 33 ± 5	Centre: 23 ± 5.4	Hooker: 0.38 ± 0.1	Centre: 0.3 ± 0.1		
				Second row: 35 ± 7	Back three: 21 ± 5.8	Second row: 0.4 ± 0.1	Back three: 0.2 ± 0.1		
				Back row: 35 ± 10		Back row: 0.4 ± 0.2			
		Load per collision						Forwards:	Backs:
								Prop: 7.9 ± 1.4	Half back: 7.6 ± 1.4
								Hooker: 7.7 ± 1.4	Centre: 8.0 ± 1.4
								Second row: 7.3 ± 1.4	Back three: 8.3 ± 1.6
								Back row: 7.6 ± 1.6	
McLaren et al. (2015) [58]	15 matches	Impacts	Total	Total: 50 ± 289		Total: 0.7 ± 0.4		NR	
				Forwards: 78 ± 18		Forwards: 1 ± 0.3			
				Backs: 28 ± 12		Backs: 1.1 ± 0.2			
Reardon et al. (2017) [24]	13 matches	Collisions	Total	Prop: 34 ± 11		NR		NR	
				Hooker: 33 ± 9					
				Second row: 35 ± 11					
				Back row: 44 ± 10					
				Scrum half: 11 ± 6					
				Out-half: 21 ± 7					
				Centre: 20 ± 5					
				Wing: 20 ± 5					
				Full back: 21 ± 6					
Takeda et al. (2014) [80]	Training and simulated match	Tackles	Total number	37.6 ± 3		NR		NR	
		Contacts		10.4 ± 2.5					
Tierney et al. (2020) [23]	Match play	Collisions	Collisions per player per game	11		NR		NR	
Tierney et al. (2021) [84]	Match play	Collision count		0.4 ± 0.1		NR		NR	
		Collision load						2.8 ± 1.1	
Venter et al. (2011) [91]	5 matches	Impacts	Total	Back row forwards: 683.4 ± 295		NR		NR	
				Outside backs: 474.3 ± 81.9					
*Rugby sevens*									
Clarke et al. (2015) [36]	3–6 matches	Impacts	Total	National: 7300 ± 2200		NR		NR	
				State: 5200 ± 2400					
Clarke et al. (2016) [37]	2 matches	Collisions	NR	Men: 35		NR		NR	
				Women: 20					
Gibson et al. (2015) [47]	3 weeks training	Tackles	Count	Week 1: 22.8 ± 10.6		NR		NR	
				Week 2: 14.6 ± 9.1					
				Week 3: 15.8 ± 5.7					
Portillo et al. (2016) [62]	5 matches	Tackle	Number/min	NR		Tackle: 0.3 ± 0.1		NR	
		Ruck				Ruck: 0.3 ± 0.1			
		Ball Carry				Ball Carry: 0.2 ± 0.1			
Suarez-Arrones et al. (2014) [78]	23 matches	Tackle	Whole match	Forwards: 7.4 ± 1.8		NR		NR	
				First half: 3.3 ± 1.3					
				Second half: 4.1 ± 1.8					
			Whole match	Backs: 4.1 ± 2.4					
				First half: 2.3 ± 1.8					
				Second half: 1.9 ± 1.4					
		Ruck	Whole match	Forwards: 1 ± 1.1					
				First half: 0.4 ± 0.5					
				Second half: 0.6 ± 0.8					
			Whole match	Backs: 0.6 ± 0.9					
				First half: 0.3 ± 0.5					
				Second half: 0.4 ± 0.5					
		Scrums		Forwards:					
				First half: 2.9 ± 0.7					
				Second half: 1 ± 0.8					

*NR* not reported

**Fig. 2:**
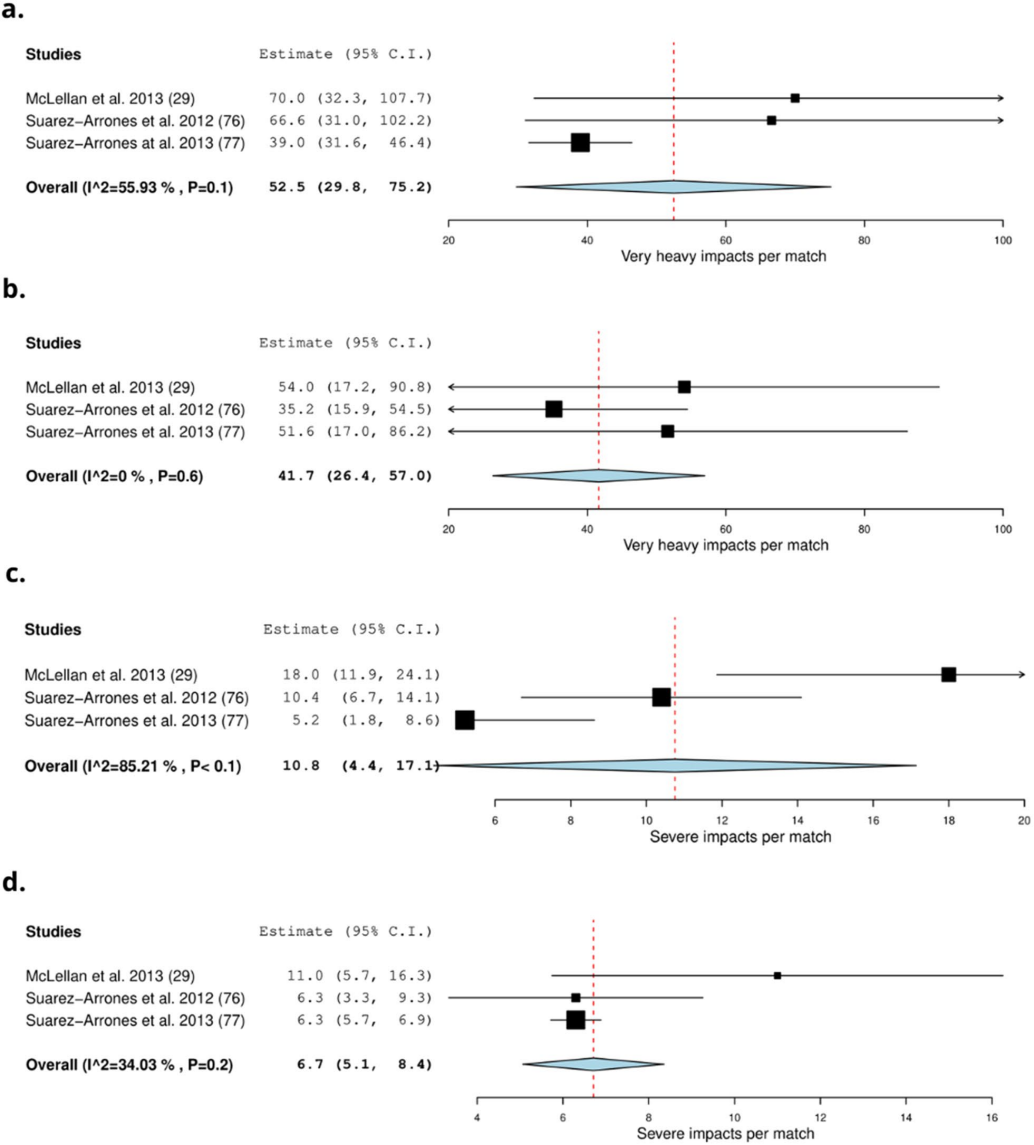
Meta-analysis of studies reporting absolute *very heavy* and *severe* impacts per match (n) from microtechnology in rugby union. The forest plot (mean and 95% confidence interval (CI)) presents the results of the meta-analysis of the pooled data estimates for the absolute *very heavy* and *severe* impact frequency for **a** forwards, **b** backs, **c** forwards and **d** backs. The squares and horizontal lines represent individual study mean and 95% CI and the diamond presents the pooled mean and 95% CI. The bigger the square the larger the sample size

**Fig. 3:**
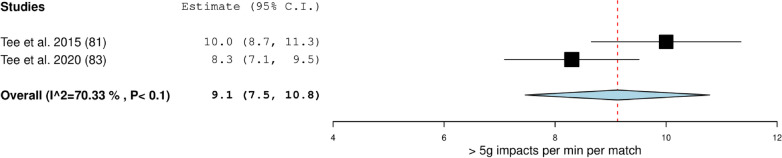
Meta-analysis of studies reporting relative > 5 g impacts frequency per match (n min^−1^) from microtechnology in rugby union. The forest plot (mean and 95% confidence interval (CI)) presents the results of the meta-analysis of the pooled data estimates for the > 5 g impacts per min per match frequency for forwards. The squares and horizontal lines represent individual study mean and 95% CI and the diamond presents the pooled mean and 95% CI. The bigger the square the larger the sample size

Sixteen studies recorded the intensity of collisions by using microtechnology (22%) (Table 3) [20, 25, 29, 39, 42, 48, 59, 61, 76, 77, 81–83, 90, 91, 95]. Forwards on average (frequency) experience 52.5 (29.8–75.2) *very heavy impacts* and 10.8 (4.4–17.1) *severe impacts* per match (Fig. 2) [29, 76, 77]. Backs experience on average 41.7 (26.4–57.0) *very heavy impacts* and 6.7 (5.1–8.4) *severe impacts* per match [29, 76, 77] (Fig. 2). Three studies recorded the relative frequency of collisions by intensity [81–83]. On average, forwards experience 9.1 (7.5–10.8) *impacts* > *5 g* per min [81, 83] (Fig. 3). Backs experience on average 9.5 (8.1–10.1) *impacts* > *5 g* per min [81, 83]. Note, Tee et al. only included > 5 g impact since it included > 8 g impacts [83]. Players experienced the highest amount of contacts in the first 20–30 min of a match and the least amount of contacts between 60 and 70 min [82]. Forwards experience more *very heavy* contacts in the second half of the match in comparison to the first half of the match. Backs experience fewer impacts in the second half of the match in comparison to the first half of the match [29]. There was no difference in impacts > 8 g per min for backs and forwards across the match [81]. Forwards experience more impacts > 5 g per min in 0–10 and 50–60 min and experienced the least amount in the 20–30 min, 40–50 min and 60–70 min intervals of the match. Backs experience more impacts > 5 g in the 0–10 min interval of the match and the 20–30 min interval of the match and the least in the 70–80 min interval [81].

**Table 3:** Characteristics of collision intensity detected by microtechnology in rugby union and rugby sevens

Study: author (year)	Type of collisions	Frequency of collisions by intensity: mean ± SD				Relative frequency of collisions by intensity: mean ± SD (no. per min)	
*Rugby union*							
Coughlan et al. (2011) [39]	Impacts	Forwards:			Backs:	NR	
		Very heavy: 53			Very Heavy: 40		
		Severe: 10			Severe: 13		
Cunniffe et al. (2009) [20]	Impacts	Forwards:			Backs:	NR	
		Very heavy: 56			Very heavy: 24		
		Severe: 13			Severe: 4		
Dubois et al. (2020) [42]	Impacts (> 8 g) weekly (game included)	Forwards:	Backs:			NR	
		23.7 ± 27	26.7 ± 38.5				
Grainger et al. (2018) [48]	Impacts	Impacts G:	Forwards:		Backs:	NR	
		Impacts > 9.01:	229 ± 160		226 ± 151		
		Impacts 9.01–11:	114 ± 79		118 ± 79		
		Impacts 11.01–13:	48 ± 41		47 ± 38		
		Impacts > 13:	66 ± 44		59 ± 40		
MacLeod et al. (2018) [25]	Impacts	Impacts (> 8 g)	Forwards:		Backs:	NR	
			Prop: 19.1 ± 7		Half back: 17.8 ± 6.9		
			Hooker: 19.6 ± 7.9		Centre: 19.1 ± 8		
			Second row: 17.7 ± 7.1		Back three: 20.4 ± 7.5		
			Back row: 18.7 ± 7.3				
McLellan et al. (2013) [29]	Impacts	Impacts (g)	Forwards:		Backs:	NR	
		Very heavy	First half: 35 ± 23		First half: 32 ± 25		
			Second half: 37 ± 25		Second half: 24 ± 19		
			Total match: 70 ± 43		Total match: 54 ± 42		
		Severe	First half: 9 ± 3		First half: 7 ± 4		
			Second half: 9 ± 6		Second half: 5 ± 4		
			Total match: 18 ± 7		Total match: 11 ± 6		
Owen et al. (2015) [59]	Impacts (first half)	Forwards:			Backs:	NR	
		Very heavy: 42 ± 21			Very Heavy: 34 ± 18		
		Severe: 25 ± 11			Severe: 22 ± 12		
		High level: 120 ± 55			High level: 99 ± 44		
Pollard et al. (2018) [61]	Collisions	NR				Mean of the whole match:	
						Forwards: 0.5 ± 0.1	
						Backs: 0.3 ± 0.1	
Suarez-Arrones et al. (2012) [76]	Impacts per match	Forwards:			Backs:	NR	
		Very heavy: 66.6 ± 48			Very Heavy: 35.2 ± 26		
		Severe: 10.4 ± 5			Severe: 6.3 ± 4		
Suarez-Arrones et al. (2013) [77]	Impacts for the match	Forwards:			Backs:	NR	
		Very heavy: 39 ± 7.6			Very heavy: 51.6 ± 35.3		
		Severe: 5.2 ± 3.5			Severe: 6.3 ± 0.6		
Tee et al. (2015) [81]	Impacts	NR				Forwards:	Backs:
						Impacts > 5G: 10 ± 3	Impacts > 5G: 9.5 ± 3.2
						Impacts > 8G: 1.1 ± 0.5	Impacts > 8G: 1.1 ± 0.4
Tee et al. (2017) [82]	Total impacts	NR				Forwards:	Backs:
						Impacts > 5G:	Impacts > 5G:
						First half: 8.7 ± 2.4	First half: 10 ± 3.5
						Q1: 9.3 ± 4.5	Q1: 10.4 ± 5.3
						Q2: 9.2 ± 2.4	Q2: 10 ± 3.9
						Q3: 8.2 ± 3.7	Q3: 10.4 ± 4.1
						Q4: 7.4 ± 2.1	Q4: 9.6 ± 4.8
						Second half: 7.9 ± 3.2	Second half: 9 ± 0.3
						Q1: 8.2 ± 3.7	Q1: 9.7 ± 3.7
						Q2: 9.4 ± 4.8	Q2: 9.4 ± 3.3
						Q3: 8.2 ± 3.1	Q3: 10 ± 3.6
						Q4: 8.7 ± 4	Q4: 7.1 ± 4
						Impacts > 8G:	Impacts > 8G:
						First half: 0.8 ± 0.3	First half: 1.1 ± 0.3
						Q1: 0.8 ± 0.6	Q1: 1 ± 0.5
						Q2: 0.9 ± 0.4	Q2: 1.1 ± 0.4
						Q3: 0.6 ± 0.3	Q3: 1.1 ± 0.4
						Q4: 0.8 ± 0.5	Q4: 1.1 ± 0.7
						Second half: 0.7 ± 0.3	Second half: 1.1 ± 0.4
						Q1: 0.8 ± 0.5	Q1: 1.1 ± 0.5
						Q2: 0.8 ± 0.4	Q2: 1.2 ± 0.6
						Q3: 0.7 ± 0.4	Q3: 1.1 ± 0.5
						Q4: 0.8 ± 0.4	Q4: 0.9 ± 0.7
Tee et al. (2020) [83]	Impacts per game (> 5 G)	NR				Forwards:	Backs:
						8.3 ± 2.7	9.5 ± 3.1
						Q1: 11 ± 5	Q1: 10 ± 4
						Q2: 8 ± 2	Q2: 10 ± 4
						Q3: 8 ± 4	Q3: 10 ± 3
						Q4: 8 ± 3	Q4: 9 ± 3
Vaz et al. (2012) [90]	Impacts	Novice:			Experienced:	NR	
		Very heavy: 21.3 ± 17.1			Very heavy: 14 ± 10.4		
		Severe: 4.7 ± 9.1			Severe: 1.6 ± 2.4		
		189.8 ± 93.3			182.5 ± 61.4		
Venter et al. (2011) [91]	Impacts	Severe impacts > 10G:				NR	
		Front row forwards: 8 ± 4.6					
		Inside backs: 12.2 ± 3.2					
Yamamoto et al. (2020) [95]	Impacts total	Impacts 8.1–10 and > 10 g: (mean ± Standard error)		Impacts 8.1–10 and > 10 g: (mean ± Standard error)		NR	
		Forwards: 202.3 ± 14.5		Backs: 171.9 ± 6.3			
		Props: 192.4 ± 17.6		Scrumhalf: 138.1 ± 31.4			
		Hooker: 197.2 ± 24.7		Fly-half: 145.9 ± 14.9			
		Locks: 225.4 ± 36		Centres: 217.9 ± 11.2			
		Flankers: 181.8 ± 11		Wings: 149.5 ± 8			
		No. 8: 196 ± 17.9		Fullback: 168.5 ± 18.9			
		Impacts > 10 g: (mean ± Standard error)		Impacts > 10 g: (mean ± Standard error)			
		Forwards: 48 ± 4.3		Backs: 35.6 ± 2.1			
		Props: 40.5 ± 7		Scrumhalf: 26.6 ± 7.6			
		Hooker: 20.5 ± 5.1		Fly-half: 35.6 ± 6			
		Locks: 57 ± 10.1		Centres: 42.4 ± 4.8			
		Flankers: 42.6 ± 3.8		Wings: 31.3 ± 2.7			
		No. 8: 50.2 ± 8.5		Fullback: 36.5 ± 5.1			
*Rugby sevens*							
Clarke et al. (2015) [35]	Impacts	Day one:		Day two:		NR	
	National: 5–6 games	Impacts 8–10 g:		Impacts 8–10 g:			
		National: 32 ± 14		National: 34 ± 24			
	State: 4–6 games	State: 26 ± 18		State: 23 ± 17			
		Impacts > 10 g:		Impacts > 10 g:			
		National: 15 ± 6		National: 17 ± 9			
		State: 12 ± 7		State: 10 ± 5			
Clarke et al. (2015) [36]	Impacts	Impacts > 10 g:				NR	
		National: 29 ± 11					
		State: 22 ± 11					
Clarke et al. (2017) [38]	Impacts	Impacts > 10 g Elite:				NR	
		Male: 25 ± 11.2					
		Female: 12.6 ± 4.7					
		Impacts > 10 g Senior:					
		Male: 11.8 ± 6.6					
		Female: 10.2 ± 7.1					
Higham et al. (2016) [51]	Impacts during the 22 matches	NR				Forwards: 26.2 ± 10.7	
						Backs: 23.5 ± 9.6	
Suarez-Arrones et al. (2014) [78]	Impacts	Forwards:			Backs:	NR	
		Very Heavy:			Very Heavy:		
		First half: 9 ± 5.1			First half: 8 ± 6.1		
		Second half: 7 ± 3.7			Second half: 6.6 ± 3.8		
		Severe:			Severe:		
		First half: 0.7 ± 1			First half: 0.9 ± 1.1		
		Second half: 1.4 ± 1.3			Second half: 1.9 ± 1.8		
		Impacts > 7 g:			Impacts > 7 g:		
		Whole match: 45.1 ± 24.5			Whole match: 41.8 ± 20.7		

*NR* not reported

#### Rugby Union Training

Two studies recorded collision frequency using microtechnology during training (3%) [32, 80]. Bradley et al. (2015) recorded the contact number of weekly training sessions of forwards and backs. Note, match data were also included in this training week [32]. Takeda et al. (2014) recorded 10.4 ± 2.5 tackles and 37.6 ± 3.0 contacts during a training simulated match [80].

#### Sevens Match-Play

**Fig. 4:**
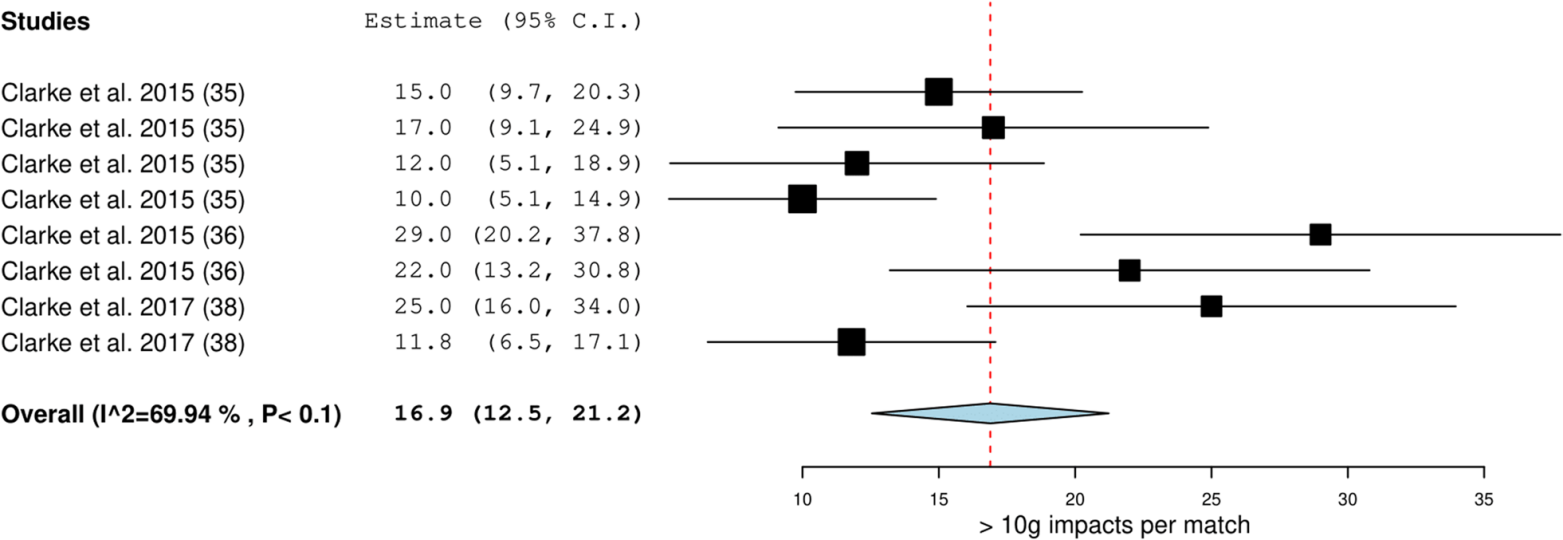
Meta-analysis of studies reporting absolute > 10 g impacts per match (*n*) from microtechnology in sevens. The forest plot (mean and 95% confidence interval (CI)) presents the results of the meta-analysis of the pooled data estimates for the absolute > 10 g impacts frequency per match. The squares and horizontal lines represent individual study mean and 95% CI and the diamond presents the pooled mean and 95% CI. The bigger the square the larger the sample size

Eight studies (11%) reported collision frequency using microtechnology during match-play [35–38, 47, 51, 62, 78]. One study reported positional groupings (forwards and backs) [78], another study reported the level of play [36] and another study reported collision frequency by sex [37] (Table 2). Collision types included impacts, collisions, tackles, rucks and scrums. Only one study recorded the relative frequency of tackles, ball carries in contact and rucks [62] and another study recorded relative frequency of impacts for forwards and backs [51]. Of the eight studies, only five reported the intensity of collisions (63%) (Table 3) [35, 36, 38, 51, 78]. Three studies recorded 16.9 (12.5–21.2) *impacts* > *10 g* per match (Fig. 4) [35, 36, 38]*.*

#### Sevens Training

Only one study reported tackle frequency during training (on average 17.8 ± 4.4 tackles per week) [47].

### Video-Based Analysis

#### Rugby Union Match-Play

**Fig. 5:**
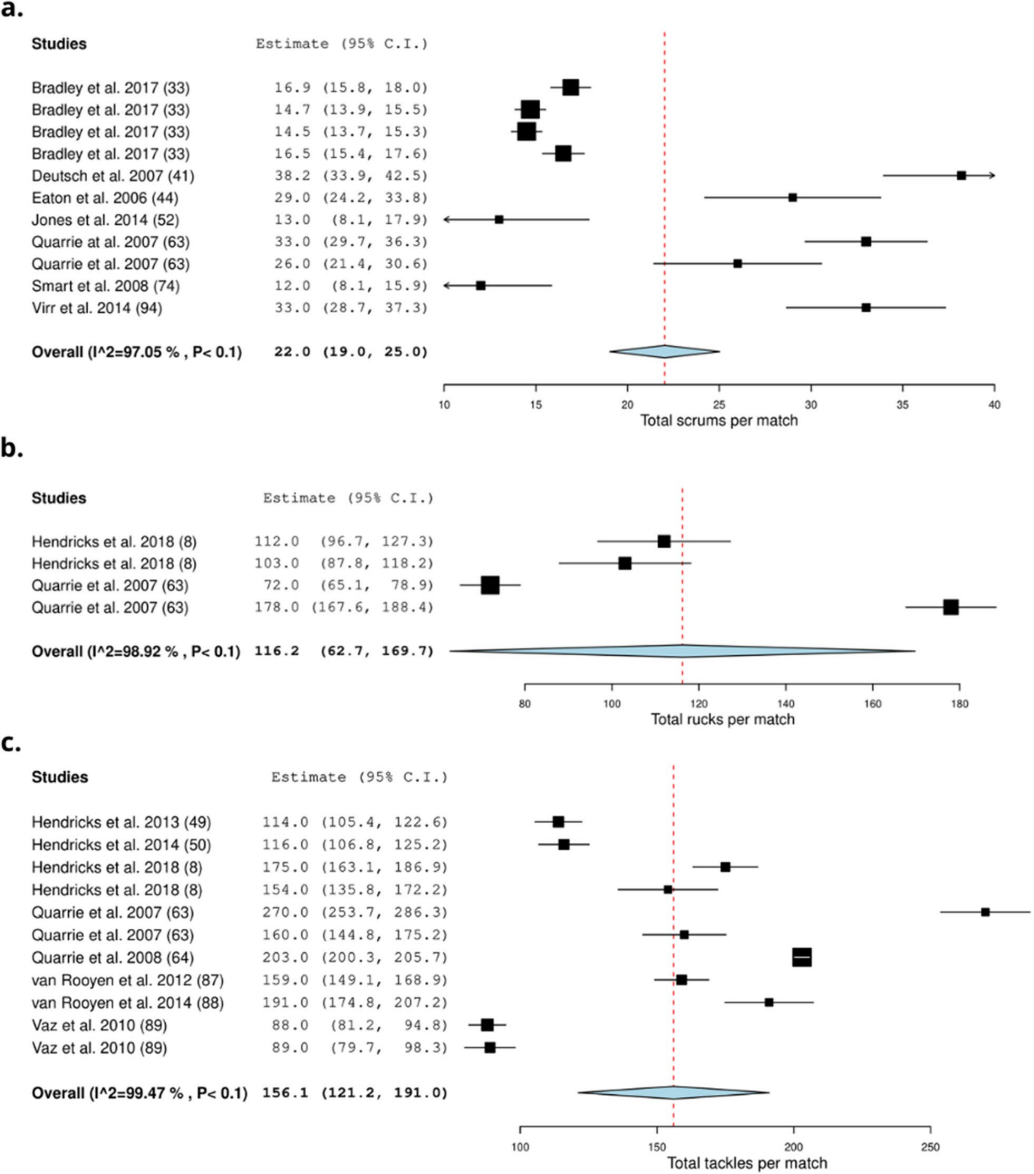
Meta-analysis of studies reporting absolute total scrums, rucks, and tackles per match (n) from video-based analysis in rugby union. The forest plot (mean and 95% confidence interval (CI)) presents the results of the meta-analysis of the pooled data estimates for the total **a** scrums, **b** rucks and **c** tackles per match. The squares and horizontal lines represent individual study mean and 95% CI and the diamond presents the pooled mean and 95% CI. The bigger the square the larger the sample size

**Fig. 6:**
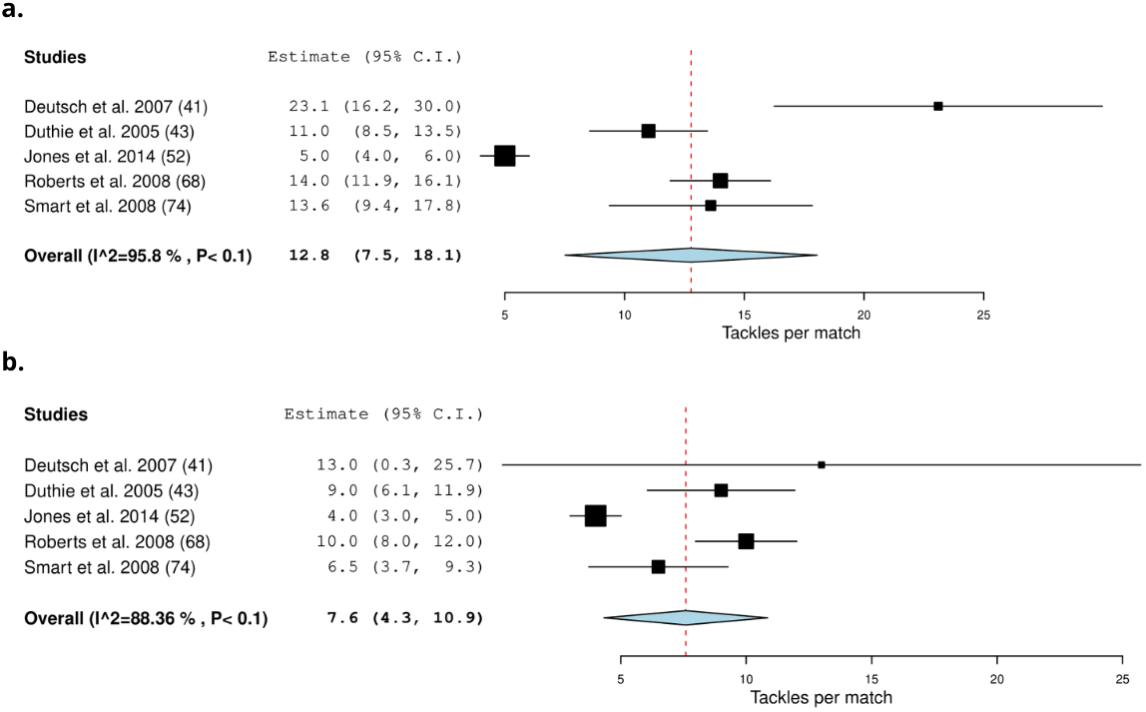
Meta-analysis of studies reporting absolute tackles per match (n) from video-based analysis in rugby union. The forest plot (mean and 95% confidence interval (CI)) presents the results of the meta-analysis of the pooled data estimates for the absolute tackle frequency for **a** forwards and **b** backs. The squares and horizontal lines represent individual study mean and 95% CI and the diamond presents the pooled mean and 95% CI. The bigger the square the larger the sample size

**Fig. 7:**
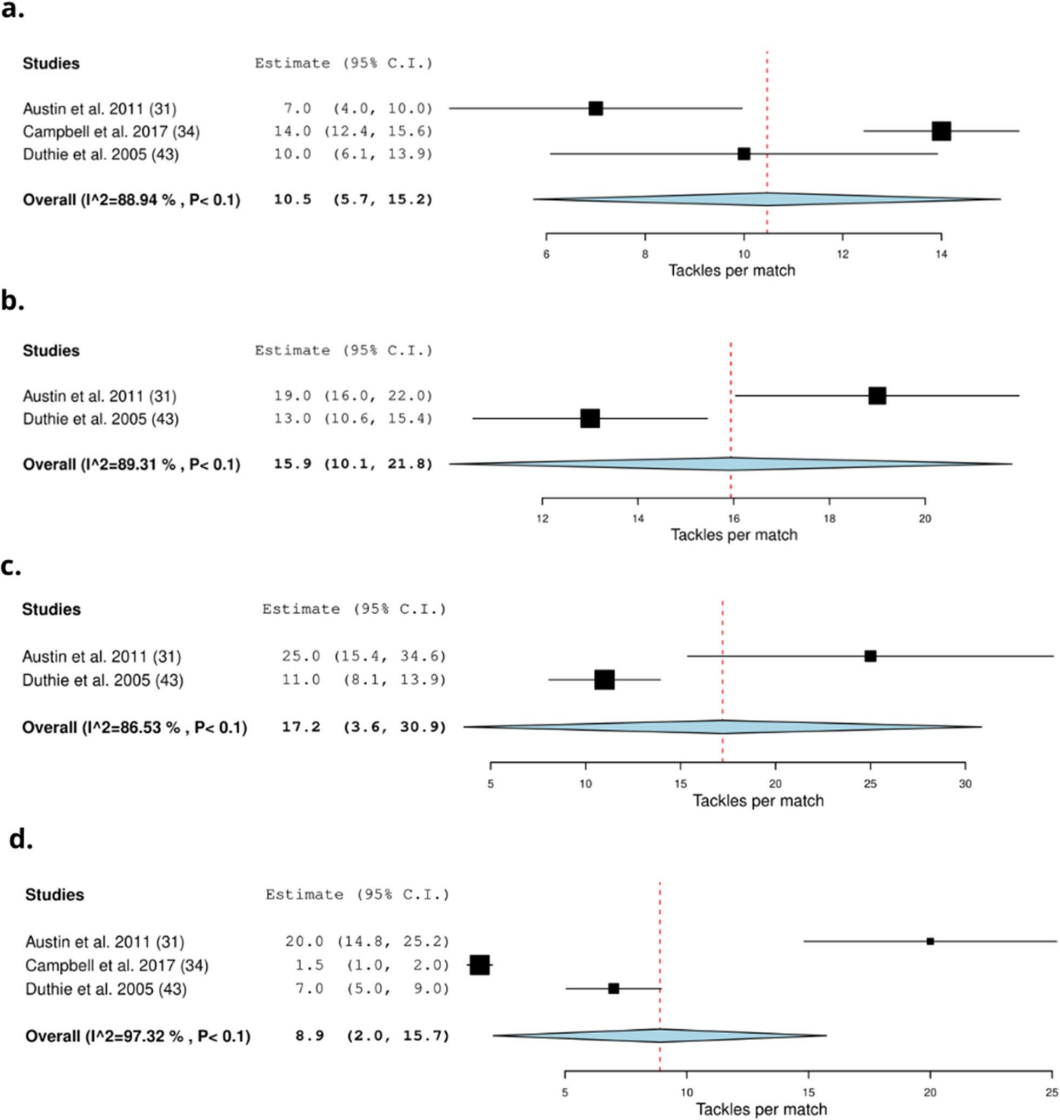
Meta-analysis of studies reporting absolute tackles per match (*n*) from video-based analysis in rugby union. The forest plot (mean and 95% confidence interval (CI)) presents the results of the meta-analysis of the pooled data estimates for the absolute tackle frequency for **a** front row forwards, **b** back row forwards, **c** inside backs and **d** outside backs. The squares and horizontal lines represent individual study mean and 95% CI and the diamond presents the pooled mean and 95% CI. The bigger the square the larger the sample size

**Fig. 8:**
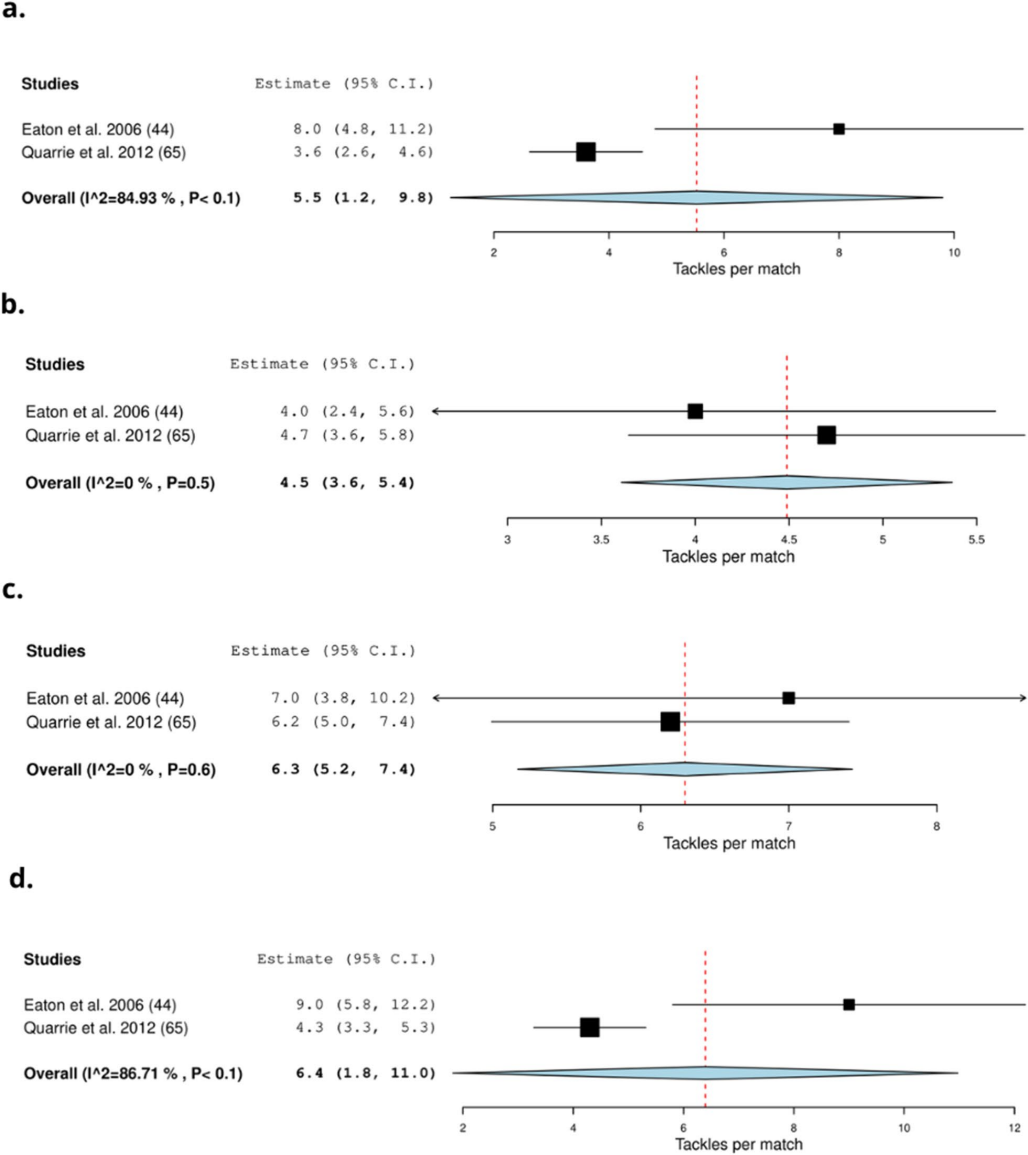
Meta-analysis of studies reporting absolute tackles per match (n) from video-based analysis in rugby union. The forest plot (mean and 95% confidence interval (CI)) presents the results of the meta-analysis of the pooled data estimates for the absolute tackle frequency for **a** props, **b** locks, **c** hooker and **d** scrumhalf. The squares and horizontal lines represent individual study mean and 95% CI and the diamond presents the pooled mean and 95% CI. The bigger the square the larger the sample size

Thirty-seven studies recorded the collision frequency using video-based analysis methods (51%) [8, 24, 31, 33, 34, 40, 41, 43–46, 49, 50, 52, 54–57, 63–66, 68, 69, 73–75, 79, 85–90, 92–94] (Table 4). Thirty-five studies were conducted during matches (95%) [8, 24, 31, 33, 40, 41, 43–46, 49, 50, 52, 54–57, 63–66, 68, 69, 73–75, 79, 85–89, 92–94], one investigated training (3%) [90] and one study investigated matches and training (3%) [34]. On average (frequency) a total of 22.0 (19.0–25.0) scrums [33, 41, 44, 52, 63, 74, 94], 116.2 (62.7–169.7) rucks [8, 63], and 156.1 [121.2–191.0] tackles occur per match (Fig. 5) [8, 49, 50, 63, 64, 87–89]. On average, forwards experience 12.8 (7.5–18.1) tackles [41, 43, 52, 68, 74] and backs experience 7.6 [4.3–10.9] tackles (Fig. 6) [41, 43, 52, 68, 74]. On average front row forwards perform 10.5 (5.7–15.2) tackles [31, 34, 43], back row forwards perform 15.9 (10.1–21.8) tackles [31, 43], inside backs perform 17.2 (3.6–30.9) tackles [31, 43] and outside backs perform 8.9 (2.0–15.7) tackles per match (Fig. 7) [31, 34, 43]. Props experience on average 5.5 [1.2–9.8] tackles per match [44, 65], locks experience 4.5 (3.6–5.4) tackles per match [44, 65], hookers experience 6.3 (5.2–7.4) tackles [44, 65] and scrumhalves experience 6.4 (1.8–11.0) tackles per match [44, 65] (Fig. 8).

**Table 4:** Characteristics of collision frequency detected by video-based analysis in rugby union and rugby sevens

Study: author (year)	Number of matches/training sessions	Type of collisions	Frequency definition	Frequency of collisions: mean ± SD		Relative frequency of collisions: mean ± SD (no. per min)	
*Rugby union*							
Austin et al. (2011) [31]	7 matches	Tackling	Number during match play	Front row forwards: 20 ± 4		NR	
				Back row forwards: 19 ± 4			
				Inside backs: 25 ± 13			
				Outside backs: 20 ± 7			
		Scrummaging (ruck/maul/scrum)		Front row forwards: 62 ± 13			
				Back row forwards: 68 ± 15			
				Inside backs: 17 ± 7			
				Outside backs: 14 ± 5			
Bradley et al. (2017) [33]	60 matches	Scrums	Scrum (count) total:	2013: 16.9 ± 4.3		NR	
				2014: 14.7 ± 3.3			
				2015: 14.5 ± 3.3			
				2016: 16.5 ± 4.5			
Campbell et al. (2017) [34]	14 matches	Tackles	Per match or training session	Match:	Training:	Match:	Training:
	29 training session		Outside backs:	1.5 ± 1	1.1 ± 1.5	0.01 ± 0.01	0.01 ± 0.01
			Centres:	5.7 ± 2.6	2.9 ± 3.1	0.06 ± 0.02	0.03 ± 0.04
			Halves:	4.5 ± 2.4	1.8 ± 2.2	0.05 ± 0.02	0.02 ± 0.02
			Loose forwards:	7.2 ± 3.2	2.4 ± 2.6	0.08 ± 0.03	0.02 ± 0.04
			Locks forwards:	6 ± 2.9	2.4 ± 2.6	0.07 ± 0.04	0.02 ± 0.02
			Front row forwards:	5.6 ± 3	1.7 ± 1.8	0.07 ± 0.05	0.02 ± 0.02
		Rucks	Loose forwards:	12.9 ± 4.2	1.3 ± 3.8	0.1 ± 0.04	0.01 ± 0.04
			Locks forwards:	15 ± 6.4	1 ± 4.1	0.2 ± 0.1	0.01 ± 0.04
			Front row forwards:	10.9 ± 4.5	1.2 ± 3.6	0.2 ± 0.1	0.01 ± 0.03
		Mauls	Loose forwards:	3.1 ± 2.7	1.5 ± 3	0.03 ± 0.03	0.01 ± 0.03
			Locks forwards:	3.3 ± 3	1.9 ± 3.3	0.03 ± 0.03	0.02 ± 0.03
			Front row forwards:	2.9 ± 2.6	1.8 ± 3.4	0.04 ± 0.04	0.02 ± 0.04
		Scrums	Loose forwards:	23.4 ± 3.9	1.8 ± 3.4	0.3 ± 0.06	0.02 ± 0.06
			Locks forwards:	21.4 ± 7.2	1.6 ± 3.2	0.3 ± 0.1	0.01 ± 0.03
			Front row forwards:	21.7 ± 5.5	1.6 ± 3.2	0.3 ± 0.2	0.01 ± 0.03
Deutsch et al. (1998) [40]	4 matches	Ruck/maul	Total	Props and Locks: 72 ± 7		NR	
				Back row: 78 ± 8			
				Inside backs: 12 ± 2			
				Outside backs: 9 ± 4			
		Scrum		Props and Locks: 32 ± 3			
				Back row: 35 ± 1			
Deutsch et al. (2007) [41]	9 matches			Forwards:	Backs:	NR	
		Ruck/maul	Total	66.9 ± 15.8	9.5 ± 5.7		
		Scrums		38.2 ± 8.7			
		Tackling		23.1 ± 14	23.4 ± 10.2		
Duthie et al. (2005) [43]	16 matches			Forwards:	Backs:	NR	
		Static exertion	No per game	Front row: 78 ± 16	Inside back: 27 ± 10		
				Back row: 82 ± 17	Outside back: 13 ± 5		
				Total: 80 ± 17	Total: 21 ± 11		
		Tackles	No per game	Front row: 10 ± 8	Inside back: 11 ± 6		
				Back row: 13 ± 5	Outside back: 7 ± 4		
				Total: 11 ± 7	Total: 9 ± 6		
Eaton et al. (2006) [44]	6 matches	Rucks and mauls	Number	Prop: 38 ± 12		NR	
				Hooker: 49 ± 10			
				Lock: 49 ± 19			
				Loose: 48 ± 13			
				Scrum half: 15 ± 5			
				Inside back: 15 ± 9			
				Outside back: 13 ± 6			
		Tackling: Tackler		Prop: 8 ± 4			
				Hooker: 8 ± 4			
				Lock: 11 ± 3			
				Loose: 13 ± 6			
				Scrum half: 11 ± 4			
				Inside back: 9 ± 4			
				Outside back: 6 ± 3			
		Tackled		Prop: 5 ± 3			
				Hooker: 7 ± 4			
				Lock: 4 ± 2			
				Loose: 8 ± 5			
				Scrum half: 9 ± 4			
				Inside back: 5 ± 3			
				Outside back: 5 ± 3			
		Scrums		Prop: 29 ± 6			
				Hooker: 29 ± 6			
				Lock: 29 ± 6			
				Loose: 27 ± 7			
			Average total	29 ± 6			
Fuller et al. (2007) [45]	50 matches	Contact events	Total	22,842		NR	
		Scrums	Total	1447			
		Tackles	Total	11,048			
		Rucks	Total	7124			
		Mauls	Total	921			
Fuller et al. (2008) [46]	26 matches	Tackles	General play total	6219		NR	
		One on one tackles	No of tackles in general play:	Tackler-1 (all): 3558			
				Arm: 1690			
				Collision: 384			
				Jersey: 93			
				Lift: 16			
				Shoulder: 826			
				Smoother: 526			
				Tap: 23			
		Double tackles	No of tackles in general play:	Tackler-1 (all): 2512			
				Arm: 1443			
				Collision: 10			
				Jersey: 86			
				Lift: 11			
				Shoulder: 746			
				Smoother: 209			
				Tap: 7			
				Tackler-2 (all): 2512			
				Arm: 1589			
				Collision: 14			
				Jersey: 22			
				Lift: 3			
				Shoulder: 358			
				Smoother: 527			
				Tap: 2			
		Arm double tackles:	No of tackles in general play:	Ball Carrier:			
				Forward: 650			
				Back: 750			
		One-on-one collision tackles:	No of tackles in general play:	Ball Carrier:			
				Forward: 146			
				Back: 217			
Hendricks et al. (2013) [49]	21 matches	Tackles	Per match	114 ± 20		NR	
		Scrums	Total	199			
		Maul	Total	152			
Hendricks et al. (2014) [50]	18 matches	Tackles	Per match	116 ± 20		NR	
			Each competition week	149			
			Per team	131			
Hendricks et al. (2018) [8]	12: Six Nations	Tackles	Total	4479		NR	
	15: Championship		Championship	1853			
			Six Nations	2626			
			Per match in Six Nations	175 ± 21			
			Per match in Championship	154 ± 36			
		Rucks	Total	2914			
			Championship	1234			
			Six Nations	1680			
			Per match in Six Nations	112 ± 27			
			Per match in Championship	103 ± 30			
Jones et al. (2014) [52]	4 matches			Forwards:	Backs:		
		Tackles	Per match	5 ± 3	4 ± 3		
		Contacts hit	Per match	15 ± 6	6 ± 4		
		Impacts	Total	25 ± 9	15 ± 7		
		Scrums	Number	13 ± 5	0		
		Contacts	Total	31 ± 14	16 ± 7		
Lacome et al. (2016) [54]	18 matches	Tackles	Players Completing Entire Match	NR		Forwards:	Backs:
						First half:	First half:
						0.1 ± 0.1	0.1 ± 0.1
						Second half: 0.1 ± 0.1	Second half: 0.1 ± 0.1
Lindsay et al. (2015) [55]	NR	Impacts:	Total	NR		Group: 0.5 ± 0.2	
						Forwards: 0.6 ± 0.2	
						Backs: 0.4 ± 0.2	
						Front row: 0.5 ± 0.1	
						Locks: 0.5 ± 0.01	
						Loose forwards: 0.6 ± 0.4	
						Inside backs: 0.4 ± 0.2	
						Outside backs: 0.3 ± 0.1	
		Tackles and tackle assists:	Total			Groups: 0.1 ± 0.1	
						Forwards: 0.2 ± 0.1	
						Backs: 0.1 ± 0.1	
						Front row: 0.1 ± 0.1	
						Locks: 0.2 ± 0.1	
						Loose forwards: 0.2 ± 0.1	
						Inside backs: 0.1 ± 0.1	
						Outside backs: 0.07 ± 0.1	
		Rucks:	Total			Groups: 0.2 ± 0.2	
						Forwards: 0.3 ± 0.3	
						Backs: 0.1 ± 0.1	
						Front row: 0.3 ± 0.1	
						Locks: 0.3 ± 0.1	
						Loose forwards: 0.4 ± 0.4	
						Inside backs: 0.2 ± 0.1	
						Outside backs: 0.1 ± 0.03	
		Ball carries	Total			Groups: 0.1 ± 0.1	
						Forwards: 0.1 ± 0.1	
						Backs: 0.1 ± 0.1	
						Front row: 0.1 ± 0.1	
						Locks: 0.1 ± 0.02	
						Loose forwards: 0.1 ± 0.1	
						Inside backs: 0.1 ± 0.1	
						Outside backs: 0.1 ± 0.1	
Lindsay et al. (2017) [56]	2 matches	Impacts	Total	Game 1: 21.3 ± 13.4		NR	
				Game 2: 26.8 ± 13.5			
McIntosh et al. (2010) [57]	77 matches (15 Elite, 15 Grade, 24 < 20)	Collisions	Total	Elite: 1422		Tackle per hour:	
				Grade: 1368		Elite: 142	
				< 20: 2000		Grade: 152	
						< 20: 135	
Quarrie et al. (2007) [63]	26 matches		Number of match activities	1995:	2004:	NR	
		Scrums		33 ± 7	26 ± 7		
		Rucks		72 ± 18	178 ± 27		
		Mauls		33 ± 8	22 ± 9		
		Tackles		160 ± 32	270 ± 25		
Quarrie et al. (2008) [64]	434 matches	Tackle events	Total analysed	140,269		NR	
			Per game	203 ± 29			
Quarrie et al. (2012) [65]	27 matches	Scrums	Per match	Prop: 25 ± 7.8		NR	
				Hooker: 25 ± 7.6			
				Lock: 25 ± 7.9			
				Flankers: 25 ± 7.9			
				Number 8: 25 ± 7.5			
		Mauls	Per match	Prop: 1.4 ± 1.5			
				Hooker: 2 ± 2.04			
				Lock: 1.9 ± 1.9			
				Flankers: 1.8 ± 1			
				Number 8: 1.8 ± 1.4			
				Scrum Half: 0.2 ± 1			
				Fly Half: 0.2 ± 0.8			
				Midfield back: 0.3 ± 0.8			
				Wing: 0.2 ± 1			
				Full back: 0.3 ± 0.8			
		Successful tackles	Per match	Prop: 7.9 ± 3.6			
				Hooker: 9.7 ± 3.8			
				Lock: 11 ± 3.8			
				Flankers: 14 ± 4.1			
				Number 8: 12 ± 4			
				Scrum Half: 8.2 ± 3.3			
				Fly Half: 9.7 ± 3.5			
				Midfield back: 10 ± 4			
				Wing: 5.5 ± 2.7			
				Full back: 4.1 ± 2.3			
		Number of times tackled	Per match	Prop: 3.6 ± 2.6			
				Hooker: 6.2 ± 3.2			
				Lock: 4.7 ± 2.8			
				Flankers: 6.1 ± 3.4			
				Number 8: 9.7 ± 3.9			
				Scrum Half: 4.3 ± 2.7			
				Fly Half: 3.9 ± 2.6			
				Midfield back: 6.5 ± 3.1			
				Wing: 5.4 ± 2.9			
				Full back: 6.1 ± 3.1			
Reardon et al. (2017) [24]	13 matches	Collisions	Total	Prop: 33 ± 8		NR	
				Hooker: 29 ± 8			
				Second row: 33 ± 7			
				Back row: 42 ± 8			
				Scrum half: 10 ± 6			
				Out half: 19 ± 3			
				Centre: 23 ± 7			
				Wing: 22 ± 3			
				Fullback: 20 ± 5			
Reardon et al. (2017) [66]	17 matches	Collisions	NR	NR		Tight five forwards: 0.7 ± 0.6–0.8	
						Back row forwards: 0.9 ± 0.8–1.01	
						Inside backs: 0.3 ± 0.2–0.4	
						Outside backs: 0.4 ± 0.3–0.6	
Roberts et al. (2008) [68]	NR			Forwards:	Backs:	NR	
		Rucks	Number	35 ± 8	11 ± 6		
		Mauls		25 ± 8	4 ± 4		
		Scrums		21 ± 12			
		Tackle		14 ± 4	10 ± 4		
Roberts et al. (2014) [69]	30 matches (10 from each group: A, B, C)	Collisions	Total analysed	370		NR	
		Scrums	Per match	32.2			
		Tackles	Per match	140.9			
		Rucks	Per match	115.0			
		Mauls	Per match	23.4			
Schoeman et al. (2015) [73]	30 matches	Tackles	Per position	60		NR	
			Total tackles in 30 games:	Loose-head prop: 568			
				Hooker: 475			
				Tight-head prop: 553			
				Loose-head lock: 666			
				Tight-head lock: 674			
				Blind-side flank: 742			
				Open-side flank: 868			
				Eighthman: 797			
				Scrum-half: 423			
				Fly-half: 505			
				Left wing: 277			
				Inside centre: 668			
				Outside centre: 515			
				Right wing: 319			
				Full-back: 301			
			Mean collision rate/80 min:	Loose-head prop: 39.3			
				Hooker: 38.5			
				Tight-head prop: 42.1			
				Loose-head lock: 44.8			
				Tight-head lock: 41.2			
				Blind-side flank: 46.1			
				Open-side flank: 50.9			
				Eighthman: 43.1			
				Scrum-half: 16.3			
				Fly-half: 19.5			
				Left wing: 19.4			
				Inside centre: 32.3			
				Outside centre: 25.7			
				Right wing: 19.9			
				Full-back: 20.5			
			Mean tackle rate/80 min:	Loose-head prop: 12.1			
				Hooker: 11.1			
				Tight-head prop: 13.2			
				Loose-head lock: 13.7			
				Tight-head lock: 14.1			
				Blind-side flank: 16.6			
				Open-side flank: 17.3			
				Eighthman: 14.7			
				Scrum-half: 8.9			
				Fly-half: 9.4			
				Left wing: 5.2			
				Inside centre: 12.9			
				Outside centre: 9.9			
				Right wing: 6.3			
				Full-back: 5.4			
Smart et al. (2008) [74]	5 matches			Forwards:	Backs:	Forwards:	Backs:
		Tackles made	Per match	13.6 ± 7.5	6.5 ± 4.7	0.6 ± 0.2	0.2 ± 0.1
		Scrums	Number	12 ± 4.4	0		
		Scrums	Total	147.4 ± 89.8	0		
		Impact	Per match	43.6 ± 18.3	13.5 ± 7.4		
		Collisions					
Smart et al. (2014) [75]	296 matches	Tackles	Successful tackles (%)	Forwards:	Backs:	NR	
				88 ± 14	80 ± 20		
Takarada (2003) [79]	2 matches	Tackle	Mean tackles per match	14 ± 7.4		NR	
Tucker et al. (2017) [85]	1516 matches	Rucks	Per match	162.9		NR	
		Mauls	Per match	10.4			
		Tackles	Per match	158			
			Tackles/player/match	Fly half: 5			
				Scrum half: 3.8			
				Centre: 5.8			
				Full back: 2.1			
				Wing: 2.7			
				Hooker: 6.9			
				Number 8: 6.4			
				Prop: 5.5			
				Lock: 6.1			
				Flanker: 7.4			
Van Rooyen et al. (2008) [86]	7 matches	Impact contacts	Average per game	Total: 386		NR	
				Forwards: 257			
				Backs: 125			
			Scrum:	Forwards: 81			
			Ruck:	Forwards: 48			
				Backs: 8			
			Maul:	Forwards: 14			
				Backs: 4.5			
Van Rooyen et al. (2012) [87]	69 matches	Tackles	Total per match	21,886 (average 159 ± 42)		NR	
			6 Nations	165 ± 28			
			Tri Nations	141 ± 24			
			RWC	156 ± 47			
Van Rooyen et al. (2014) [88]	15 matches	Tackle	Tackle situations per match	Average: 191 ± 32		NR	
				Average winning team: 89 ± 30			
				Average losing team: 101 ± 24			
Vaz et al. (2010) [89]	**IRB competitions:** 64 matches	Tackles made:	Total	Winners:	Losers:	NR	
				88 ± 27.6	89 ± 37.8		
Vaz et al. (2012) [90]	Training session (Small sided games)	Tackles	Tackles made:	Novice:	Experienced:	NR	
				28.2 ± 3.3	48.7 ± 3.3		
Villarejo et al. (2013) [92]	48 matches	Tackles	Attempted tackles	Front row: 10		NR	
				Second row: 10.9			
				Back row: 14.3			
				Scrum halves: 12.5			
				Middle backs: 10.5			
				Back three: 5.9			
			Tackles made	Front row: 8			
				Second row: 8.6			
				Back row: 11.2			
				Scrum halves: 8.3			
				Middle backs: 7.2			
				Back three: 3.7			
			Ineffective tackles	Front row: 0.7			
				Second row: 0.6			
				Back row: 1.1			
				Scrum halves: 1.7			
				Middle backs: 1.2			
				Back three: 0.9			
Villarejo et al. (2015) [93]	48 matches	Tackles	Attempted tackles	Winning team:	Losing team:	NR	
				Front row: 10.5 ± 14.04	Front row: 9.4 ± 12.4		
				Second row: 10.2 ± 8.6	Second row: 11.6 ± 14.9		
				Back row: 14.5 ± 14.6	Back row: 14.2 ± 17.6		
				Scrum halves: 9.5 ± 11.1	Scrum halves: 15.3 ± 24.7		
				Inside backs: 9.3 ± 12.9	Inside backs: 11.4 ± 10.6		
				Outside backs: 5.5 ± 9.6	Outside backs: 6.2 ± 7.4		
			Effective tackles:	Front row: 8.9 ± 12.9	Front row: 6.8 ± 9.8		
				Second row: 8.4 ± 7.3	Second row: 8.7 ± 9.5		
				Back row: 12 ± 11.6	Back row: 10.6 ± 14.9		
				Scrum halves: 7.5 ± 9.3	Scrum halves: 8.8 ± 15.4		
				Inside backs: 7.02 ± 10.9	Inside backs: 7.1 ± 7.2		
				Outside backs: 4 ± 7.5	Outside backs: 3.3 ± 3.7		
			Ineffective tackles:	Front row: 0.5 ± 2	Front row: 0.9 ± 2.4		
				Second row: 0.5 ± 1.1	Second row: 0.8 ± 1.5		
				Back row: 1 ± 4.1	Back row: 1.1 ± 2.8		
				Scrum halves: 1.1 ± 3.1	Scrum halves: 2.3 ± 6		
				Inside backs: 0.7 ± 2.03	Inside backs: 1.5 ± 2.8		
				Outside backs: 0.5 ± 1.7	Outside backs: 1.4 ± 6.1		
Virr et al. (2014) [94]	10 matches	Ruck/maul/tackle	Total number	Forwards:	Backs:	NR	
		Scrums		61 ± 12	25 ± 11		
				33 ± 7			
*Rugby sevens*							
Clarke et al. (2016) [37]	2 matches	Collisions	Collisions	Men: 51		NR	
				Women: 44			
Hendricks et al. (2019) [3]	135 matches	Tackles	Per match	1.9 ± 1.3		NR	
			Total	8.4 ± 4.1			
		Ruck	Total	0.4 ± 0.7			
Higham et al. (2014) [5]	196 matches	Scrums	Per team per match	1.9 ± 0.1		NR	
		Rucks	Per team per match	8.4 ±.0.6			
Peeters et al. (2019) [60]	32 matches	Contact actions	Tackles/collisions/rucks/ mauls	Forwards:	Backs:	NR	
				First half: 5.3 ± 2.8	First half: 5.3 ± 3		
				Second half: 6.3 ± 2.9	Second half: 6.1 ± 2.7		
Reyneke et al. (2018) [67]	15 matches	Tackles:	Low (< 21 score):	3.4 ± 1.8		NR	
			High (>/ = 21 score):	3 ± 2			
		Scrums	Low (< 21 score):	1.6 ± 1.3			
			High (>/ = 21 score):	1.2 ± 1.8			
		Ball Carry	Low (< 21 score):	4.4 ± 2.9			
			High (>/ = 21 score):	4.9 ± 2.5			
Ross et al. (2015) [70]	NR	Tackles:	**Total**	NR			
			Provincial:			0.2 ± 0.1	
			International:			0.2 ± 0.2	
		Rucks:	Provincial:			0.1 ± 0.1	
			International:			0.2 ± 0.2	
		Ball Carries:	Provincial:			0.3 ± 0.2	
			International:			0.2 ± 0.2	
Ross et al. (2015) [71]	54 matches			Forwards:	Backs:	NR	
		Tackles	Per match	2.7 ± 2.6	2.41 ± 2.5		
		Scrums		1.8 ± 1.9			
		Ball Carries		3.2 ± 2.4	4.1 ± 3.2		
Ross et al. (2016) [72]	37 matches (between team analysis)	Tackles	Dominant tackles per match:	2.1 ± 2.3		NR	
	50 matches (single team analysis)		Ineffective tackles:	8.1 ± 3.9			
		Rucks	Defensive ruck average per match:	1.2 ± 0.3			
			Ruck average:	1.2 ± 0.2			

*NR* not reported, *RWC* Rugby World Cup

#### Rugby Union Training

Only one study reported collision frequency during training [90]. Vaz et al. (2012) reported that novice players perform an average of 28.2 ± 3.3 tackles during small-sided games, while experienced players perform 48.7 ± 3.3 tackles on average [90].

#### Sevens Match Play

**Fig. 9:**
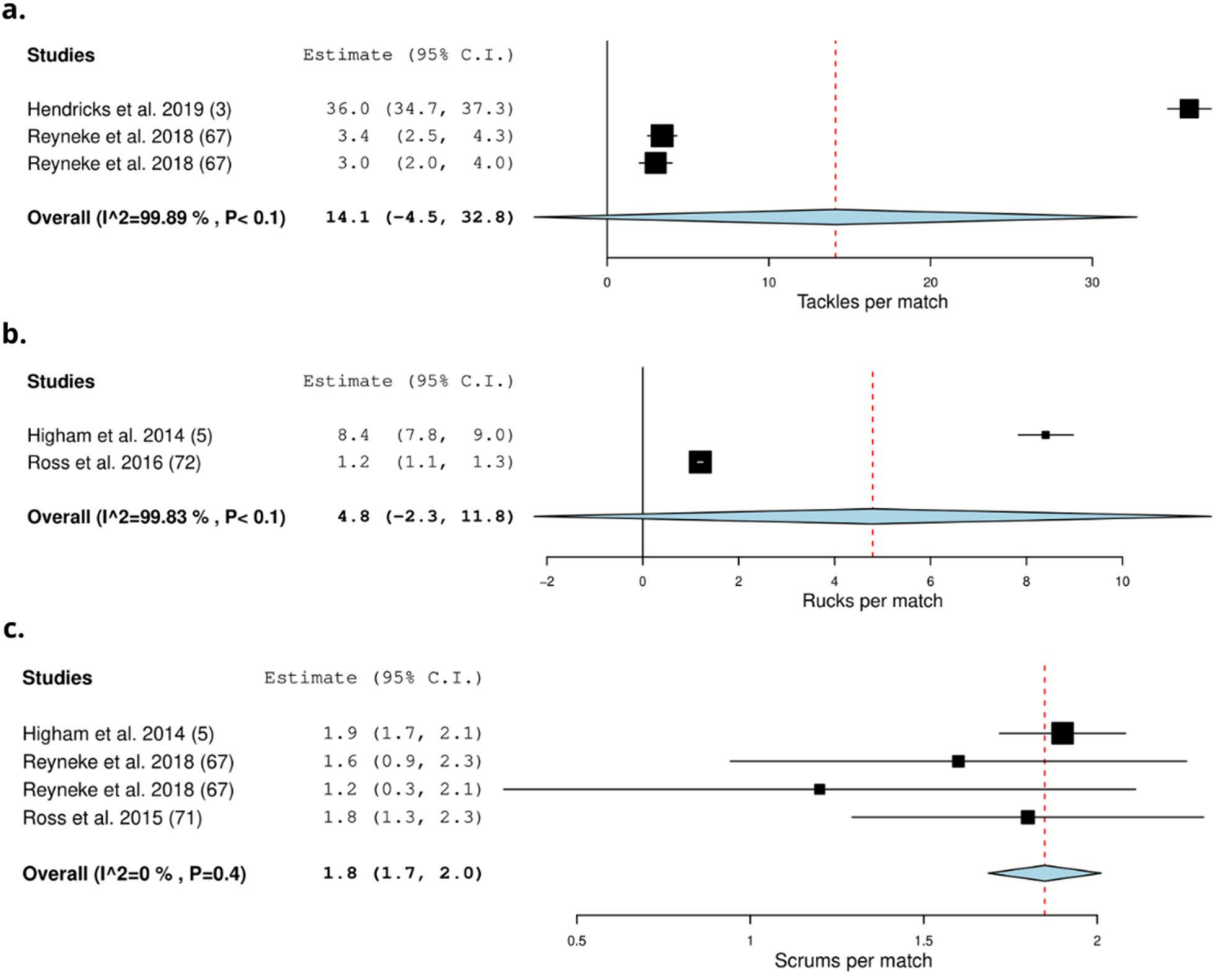
Meta-analysis of studies reporting absolute tackles, rucks, and scrums per match (n) from video-based analysis in sevens. The forest plot (mean and 95% confidence interval (CI)) presents the results of the meta-analysis of the pooled data estimates for the absolute frequency of **a** tackles, **b** rucks and **c** scrums per match. The squares and horizontal lines represent individual study mean and 95% CI and the diamond presents the pooled mean and 95% CI. The bigger the square the larger the sample size

Eight studies recorded the collision frequency by using video-based analysis (11%) (Table 4) [3, 5, 37, 60, 67, 70–72]. Ross et al. (2015) recorded the relative frequency of rucks and tackles at provincial and international level [70]. Three studies recorded the frequency of collisions [37], contact actions [60], tackles, being tackled (ball-carrier) and scrums (in relation to high and low scoring matches) [67]. Clarke et al. (2016) recorded 51 collisions for males and 44 collisions for females in a single match [37]. On average, 14.1 (0–32.8) tackles occur per match [3, 67], 4.8 (0–11.8) rucks per match [5, 72] and 1.8 (1.7–2.0) scrums per match [5, 67, 71] (Fig. 9). Finally, backs and forwards experience more contacts in the second half of the match compared to the first half [60].

#### Sevens Training

No video-based training studies were found for sevens.

## Discussion

To our knowledge, this is the first systematic review on quantifying collision frequency and intensity in rugby union and rugby sevens. This review demonstrates that video-based analysis and microtechnology are the main methods used to quantify collisions in rugby union and sevens. Not surprisingly, the absolute collision frequency during sevens matches was lower than rugby union due to the shorter duration of the game and fewer players on the field. When comparing relative frequencies though, rugby union players seem to perform less tackles and ball carries into contact than sevens players, while rucks per minute were similar between the two rugby codes [55, 70]. Expressing collision frequencies relative to playing time provides coaches and players with the ‘collision density’ [96], a metric that can potentially be used in training to better prepare players for the collision demands of matches. With that said, only two studies expressed collisions or contact events per minute in sevens [62, 70], which highlights an area for further work. In rugby union match-play, forwards experience more tackles than backs (12.8 (7.5–18.1) tackles and 7.6 (4.3–10.9) tackles, respectively). Another key finding of this review is that forwards experience more *very heavy* impacts (52.5 (29.8–75.2) vs. 41.7 (26.4–57.0) *very heavy* impacts) and *severe* impacts (10.8 (4.4–17.1) vs. 6.7 (5.1–8.4) *severe impacts*) than backs in rugby union. Coaches are recommended to train players specific to their positional grouping for appropriate adaptations. In both rugby cohorts, only six studies were completed on females [35, 36, 62, 67, 77, 94] and two studies on both sexes [37, 38]. Overall, there was a lack of consistency on the definition of a collision. Also, grouping variables (i.e., how the positions were grouped) made it hard to make comparisons. It is recommended to integrate microtechnology and video-based analysis simultaneously to ensure maximal accuracy of metrics. Given the high injury incidence and burden of collision events, it is important that we adequately prepare athletes for collisions in training to meet the collision demands of matches.

To optimise training, researchers, trainers and sport practitioners typically study competition activities and demands, and attempt to replicate these demands in training [76, 78, 93, 97]. Training is subsequently monitored to ensure athletes meet said competition activities and demands [34]. Monitoring training also ensures athletes are not exposed to any unnecessary injury risks, and are positively adapting to training [34]. Only four studies quantified collision frequencies and/or intensities in training—three in rugby union [32, 80, 90] and one in sevens [47], while 66 studies quantified frequencies and/or intensities of collisions in matches. Three studies related the frequency and intensity of collisions during training to matches—two in rugby union [34, 42] and one in sevens [51]. In both studies, collision frequencies and intensities were lower in training, suggesting that players may not be adequately preparing for matches [34, 51]. Indeed, the adaptations for a “collision-fit” player are likely to respond to general training principles including the concept of periodization [98]. Using general training concepts, such as periodisation, and collision demands data from match-play, coaches and practitioners can develop training programmes to enhance players’ adaptability and capacity to repeatably engage in physical-technical contests without increasing their risk of injury; in other words, building a ‘collision-fit’ player. Recently, this has been suggested for skill training and Hendricks et al. (2018) described such a periodised plan for the rugby tackle [99]. Understanding the adaptations for a “collision-fit” player will also allow for safer return to play protocols for collision sport athletes and reduce the risk of re-injury. To inform collision preparation practice, more work on collision training and its relationship to match demands, player development, performance and/or (re)injury risk is required. Collision training studies of this nature should also ideally be collected over more than one season and from multiple teams.

Collision frequency and intensities have been quantified in studies using video-based analysis (*n* = 37), microtechnology (*n* = 24) or both methods (*n* = 12). Each method has its advantages and disadvantages. For example, video-based analysis is laborious and reliant on human observation, while it may capture more contextual detail of the collision event [16]. Conversely, microtechnology may be more efficient and objective, but its reliability and validity for quantifying collision demands is inconclusive at this stage [16, 24, 25]. Also, customised algorithms detect collisions, making study comparisons difficult [100]. With that said, studies are emerging to support collision metrics when used in conjunction with video-based analysis [23, 25]. Although some literature supports the use of microtechnology for collision monitoring, there is still a lack of validity regarding other metrics and therefore more investigation is needed [23]. As such, a superior approach to quantifying collision demands from a research and practitioner perspective may be to integrate video and microtechnology [18, 19]. Using both video and microtechnology, coaches, practitioners and researchers are able to cross check the microtechnology data with video, determine its accuracy and distinguish between collision events [18, 24, 25].

If the goal is to ensure players are well-prepared for matches by providing the optimal collision frequency and intensity dose, the metrics (i.e., collisions, contacts, scrums, tackles, rucks and mauls) and grouping variables (i.e., specific positions, forwards and backs) between training and matches need to be consistent and more accurate. In other words, how collision demands are reported for matches should be useful to the coach and practitioner, and transferable to a training setting. Therefore, metrics and grouping variables between the two settings need to be consistent to ensure this transfer. Strong engagement with the coach and practitioner when developing reporting metrics is therefore recommended [101]. Recently, a consensus document for the video-based analysis of contact events was published to improve the consistency and quality of video-based analysis work in rugby union and sevens [18]. A similar consensus-based approach may be required for microtechnology collision metrics [16, 22]. As mentioned, many studies report collisions differently, making study comparisons difficult between groups, methods used and between rugby cohorts. As a result, this limited the current synthesis. Collision intensity metrics in particular were inconsistent between studies. The lack of consistency between studies is a key factor limiting our understanding of collision loads [16]. Additionally, the intensity of collisions is difficult to compare longitudinally, given that technology is constantly evolving. More recent technology is likely more accurate as algorithms are improved over time ensuring MEMs have a high specificity and sensitivity, and are more likely to detect a collision when it occurs [23], although limited studies can confirm this [25].

The purpose of this review was to synthesise the frequency and intensity of collisions during training and matches in rugby union and sevens. In both rugby cohorts, future studies should investigate training in comparison to match-play. Additionally, future studies should explore women’s rugby. Many of these groups were understudied and are very important in our rugby community. A consensus-based approach for microtechnology is warranted since grouping variables and metrics were inconsistent throughout the studies. Beyond this, there are a number of other factors that can affect how players respond and adapt to different frequencies and intensities of contact. Collision events in rugby union and sevens are dynamic and have a major technical-skill component [102, 103]. The opposing players’ technical ability may also affect the perceived intensity of the collision event. The perceived physical and technical demands of collision events can also be captured using subjective ratings such as rating of perceived exertion (RPE) [104] and rating of perceived challenge (RPC) [98, 104], respectively. These subjective ratings are useful when planning and monitoring training [104]. Also, collisions are interspersed between periods of high intensity running (sprinting, accelerations, decelerations) and low-intensity activities (walking, jogging). As such, advanced collision training should also include periods of high-intensity running to mimic complete match demands and fatigue conditions [97].

## Conclusion

In conclusion, this review found a discrepancy in the number of studies quantifying collision demands in training compared to matches. While more work on quantifying the collision demands of training is required, studies should also compare training and matches if we are to improve our understanding of the relationship between training and matches. Another key finding is that the main method for quantifying collisions was video-based analysis. To improve the relationship between matches and training, integrating both video-based analysis and microtechnology is recommended, and the metrics and grouping variables between training and matches should be consistent. Per minute, rugby sevens players perform more tackles and ball carries into contact than rugby union players and forwards experienced more tackles than backs (12.8 (7.5–18.1) tackles and 7.6 (4.3–10.9) tackles, respectively). Another key finding in this review is that forwards experience more very heavy impacts (52.5 (29.8–75.2) vs. 41.7 (26.4–57.0) *very heavy* impacts) and severe impacts (10.8 (4.4–17.1) vs. 6.7 (5.1–8.4) *severe* impacts) than backs in rugby union. The frequency and intensity of collisions in training and matches may lead to adaptations for a “collision-fit” player and lend themselves to general training principles such as periodisation for optimum collision adaptation. Subjective measures such as RPE and RPC should be incorporated into the monitoring and management of the collision section of training to understand the internal load.

## Supplementary Information

**Additional file 1: Table S1.** Methodological quality assessment of the final full text articles according to Downs et al. [30]. **Table S2.** Characteristics of studies using microtechnology to record collisions during match-play or training sessions. **Table S3.** Characteristics of studies using video-based analysis to record collisions during match-play or training sessions.

## Data Availability

Not applicable.
