# Correction: Quantifying Collision Frequency and Intensity in Rugby Union and Rugby Sevens: A Systematic Review

**DOI:** 10.1186/s40798-022-00494-z

**Published:** 2022-07-29

**Authors:** Lara Paul, Mitchell Naughton, Ben Jones, Demi Davidow, Amir Patel, Mike Lambert, Sharief Hendricks

**Affiliations:** 1 Division of Physiological Sciences, Department of Human Biology, Faculty of Health Sciences, University of Cape Town, Cape Town, South Africa; 2 School of Health and Behavioural Sciences, University of the Sunshine Coast, Sippy Downs, QLD Australia; 3 Centre for Human Factors and Sociotechnical Systems, University of the Sunshine Coast, Sippy Downs, QLD Australia; 4 School of Science and Technology, University of New England, Armidale, NSW Australia; 5 Carnegie Applied Rugby Research (CARR) Centre, Carnegie School of Sport, Leeds Beckett University, Leeds, UK; 6 Leeds Rhinos Rugby League Club, Leeds, UK; 7 England Performance Unit, The Rugby Football League, Leeds, UK; 8 Health Through Physical Activity, Lifestyle and Sport Research Centre (HPALS), Department of Human Biology, Faculty of Health Sciences, University of Cape Town, Cape Town, South Africa; 9 Department of Electrical Engineering, African Robotics Unit, University of Cape Town, Western Cape, South Africa

## Correction to: Sports Medicine - Open (2022) 8:12 10.1186/s40798-021-00398-4

The following errors are noted and corrected:

1. In Abstract, Results, sentence 5: ‘156.1 (121.2–191.0)’ should have been ‘171.2 (140.5–201.8)’.
2. In section Microtechnology, Rugby Union Training, final sentence: ‘contacts’ should have been ‘tackles’ and vice versa.
3. In section Video-Based Analysis, Rugby Union Match Play, sentence 3: ‘156.1 (121.2–191.0)’ should have been ‘171.2 (140.5–201.8)’.
4. In Table 4: The following additional data have been added to the Vaz et al. (2010) (89) row:
  Column 2: S12 competition: 95 matches;
  Column 5: 112.7 ± 33.1;
  Column 6: 99.4 ± 3.0.

The original version of Table 4 has been replaced with the version shown below:

**Table 4:** Characteristics of collision frequency detected by video-based analysis in rugby union and rugby sevens.

Study: author (year)	Number of matches/training sessions	Type of collisions	Frequency definition	Frequency of collisions: mean ± SD		Relative frequency of collisions: mean ± SD (no. per min)	
*Rugby union*							
Austin et al. (2011) [31]	7 matches	Tackling	Number during match play	Front row forwards: 20 ± 4		NR	
				Back row forwards: 19 ± 4			
				Inside backs: 25 ± 13			
				Outside backs: 20 ± 7			
		Scrummaging (ruck/maul/scrum)		Front row forwards: 62 ± 13			
				Back row forwards: 68 ± 15			
				Inside backs: 17 ± 7			
				Outside backs: 14 ± 5			
Bradley et al. (2017) [33]	60 matches	Scrum	Scrum (count) total:	2013: 16.9 ± 4.3		NR	
				2014: 14.7 ± 3.3			
				2015: 14.5 ± 3.3			
				2016: 16.5 ± 4.5			
Campbell et al. (2017) [34]	14 matches	Tackles	Per match or training session	Match:	Training:	Match:	Training:
	29 training session		Outside backs:	1.5 ± 1	1.1 ± 1.5	0.01 ± 0.01	0.01 ± 0.01
			Centres:	5.7 ± 2.6	2.9 ± 3.1	0.06 ± 0.02	0.03 ± 0.04
			Halves:	4.5 ± 2.4	1.8 ± 2.2	0.05 ± 0.02	0.02 ± 0.02
			Loose forwards:	7.2 ± 3.2	2.4 ± 2.6	0.08 ± 0.03	0.02 ± 0.04
			Locks forwards:	6 ± 2.9	2.4 ± 2.6	0.07 ± 0.04	0.02 ± 0.02
			Front row forwards:	5.6 ± 3	1.7 ± 1.8	0.07 ± 0.05	0.02 ± 0.02
		Rucks	Loose forwards:	12.9 ± 4.2	1.3 ± 3.8	0.1 ± 0.04	0.01 ± 0.04
			Locks forwards:	15 ± 6.4	1 ± 4.1	0.2 ± 0.1	0.01 ± 0.04
			Front row forwards:	10.9 ± 4.5	1.2 ± 3.6	0.2 ± 0.1	0.01 ± 0.03
		Mauls	Loose forwards:	3.1 ± 2.7	1.5 ± 3	0.03 ± 0.03	0.01 ± 0.03
			Locks forwards:	3.3 ± 3	1.9 ± 3.3	0.03 ± 0.03	0.02 ± 0.03
			Front row forwards:	2.9 ± 2.6	1.8 ± 3.4	0.04 ± 0.04	0.02 ± 0.04
		Scrums	Loose forwards:	23.4 ± 3.9	1.8 ± 3.4	0.3 ± 0.06	0.02 ± 0.06
			Locks forwards:	21.4 ± 7.2	1.6 ± 3.2	0.3 ± 0.1	0.01 ± 0.03
			Front row forwards:	21.7 ± 5.5	1.6 ± 3.2	0.3 ± 0.2	0.01 ± 0.03
Deutsch et al. (1998) [40]	4 matches	Ruck/maul	Total	Props and Locks: 72 ± 7		NR	
				Back row: 78 ± 8			
				Inside backs: 12 ± 2			
				Outside backs: 9 ± 4			
		Scrum		Props and Locks: 32 ± 3			
				Back row: 35 ± 1			
Deutsch et al. (2007) [41]	9 matches			Forwards:	Backs:	NR	
		Ruck/maul	Total	66.9 ± 15.8	9.5 ± 5.7		
		Scrum		38.2 ± 8.7			
		Tackling		23.1 ± 14	23.4 ± 10.2		
Duthie et al. (2005) [43]	16 matches			Forwards:	Backs:	NR	
		Static exertion	No per game	Front row: 78 ± 16	Inside back: 27 ± 10		
				Back row: 82 ± 17	Outside back: 13 ± 5		
				Total: 80 ± 17	Total: 21 ± 11		
		Tackles	No per game	Front row: 10 ± 8	Inside back: 11 ± 6		
				Back row: 13 ± 5	Outside back: 7 ± 4		
				Total: 11 ± 7	Total: 9 ± 6		
Eaton et al. (2006) [44]	6 matches	Rucks and mauls	Number	Prop: 38 ± 12		NR	
				Hooker: 49 ± 10			
				Lock: 49 ± 19			
				Loose: 48 ± 13			
				Scrum half: 15 ± 5			
				Inside back: 15 ± 9			
				Outside back: 13 ± 6			
		Tackling: Tackler		Prop: 8 ± 4			
				Hooker: 8 ± 4			
				Lock: 11 ± 3			
				Loose: 13 ± 6			
				Scrum half: 11 ± 4			
				Inside back: 9 ± 4			
				Outside back: 6 ± 3			
		Tackled		Prop: 5 ± 3			
				Hooker: 7 ± 4			
				Lock: 4 ± 2			
				Loose: 8 ± 5			
				Scrum half: 9 ± 4			
				Inside back: 5 ± 3			
				Outside back: 5 ± 3			
		Scrums		Prop: 29 ± 6			
				Hooker: 29 ± 6			
				Lock: 29 ± 6			
				Loose: 27 ± 7			
			Average total	29 ± 6			
Fuller et al. (2007) [45]	50 matches	Contact events	Total	22,842		NR	
		Scrums	Total	1447			
		Tackles	Total	11,048			
		Rucks	Total	7124			
		Mauls	Total	921			
Fuller et al. (2008) [46]	26 matches	Tackles	General play total	6219		NR	
		One on one tackles	No of tackles in general play:	Tackler-1 (all): 3558			
				Arm: 1690			
				Collision: 384			
				Jersey: 93			
				Lift: 16			
				Shoulder: 826			
				Smoother: 526			
				Tap: 23			
		Double tackles	No of tackles in general play:	Tackler-1 (all): 2512			
				Arm: 1443			
				Collision: 10			
				Jersey: 86			
				Lift: 11			
				Shoulder: 746			
				Smoother: 209			
				Tap: 7			
				Tackler-2 (all): 2512			
				Arm: 1589			
				Collision: 14			
				Jersey: 22			
				Lift: 3			
				Shoulder: 358			
				Smoother: 527			
				Tap: 2			
		Arm double tackles:	No of tackles in general play:	Ball Carrier:			
				Forward: 650			
				Back: 750			
		One-on-one collision tackles:	No of tackles in general play:	Ball Carrier:			
				Forward: 146			
				Back: 217			
Hendricks et al. (2013) [49]	21 matches	Tackles	Per match	114 ± 20		NR	
		Scrum	Total	199			
		Maul	Total	152			
Hendricks et al. (2014) [50]	18 matches	Tackles	Per match	116 ± 20		NR	
			Each competition week	149			
			Per team	131			
Hendricks et al. (2018) [8]	12: Six Nations	Tackles	Total	4479		NR	
	15: Championship		Championship	1853			
			Six Nations	2626			
			Per match in Six Nations	175 ± 21			
			Per match in Championship	154 ± 36			
		Rucks	Total	2914			
			Championship	1234			
			Six Nations	1680			
			Per match in Six Nations	112 ± 27			
			Per match in Championship	103 ± 30			
Jones et al. (2014) [52]	4 matches			Forwards:	Backs:		
		Tackles	Per match	5 ± 3	4 ± 3		
		Contacts hit	Per match	15 ± 6	6 ± 4		
		Impacts	Total	25 ± 9	15 ± 7		
		Scrum	Number	13 ± 5	0		
		Contacts	Total	31 ± 14	16 ± 7		
Lacome et al. (2016) [54]	18 matches	Tackles	Players Completing Entire Match	NR		Forwards:	Backs:
						First half:	First half:
						0.1 ± 0.1	0.1 ± 0.1
						Second half: 0.1 ± 0.1	Second half: 0.1 ± 0.1
Lindsay et al. (2015) [55]	NR	Impacts:	Total	NR		Group: 0.5 ± 0.2	
						Forwards: 0.6 ± 0.2	
						Backs: 0.4 ± 0.2	
						Front row: 0.5 ± 0.1	
						Locks: 0.5 ± 0.01	
						Loose forwards: 0.6 ± 0.4	
						Inside backs: 0.4 ± 0.2	
						Outside backs: 0.3 ± 0.1	
		Tackles and tackle assists:	Total			Groups: 0.1 ± 0.1	
						Forwards: 0.2 ± 0.1	
						Backs: 0.1 ± 0.1	
						Front row: 0.1 ± 0.1	
						Locks: 0.2 ± 0.1	
						Loose forwards: 0.2 ± 0.1	
						Inside backs: 0.1 ± 0.1	
						Outside backs: 0.07 ± 0.1	
		Rucks:	Total			Groups: 0.2 ± 0.2	
						Forwards: 0.3 ± 0.3	
						Backs: 0.1 ± 0.1	
						Front row: 0.3 ± 0.1	
						Locks: 0.3 ± 0.1	
						Loose forwards: 0.4 ± 0.4	
						Inside backs: 0.2 ± 0.1	
						Outside backs: 0.1 ± 0.03	
		Ball carries	Total			Groups: 0.1 ± 0.1	
						Forwards: 0.1 ± 0.1	
						Backs: 0.1 ± 0.1	
						Front row: 0.1 ± 0.1	
						Locks: 0.1 ± 0.02	
						Loose forwards: 0.1 ± 0.1	
						Inside backs: 0.1 ± 0.1	
						Outside backs: 0.1 ± 0.1	
Lindsay et al. (2017) [56]	2 matches	Impacts	Total	Game 1: 21.3 ± 13.4		NR	
				Game 2: 26.8 ± 13.5			
McIntosh et al. (2010) [57]	77 matches (15 Elite, 15 Grade, 24 < 20)	Collisions	Total	Elite: 1422		Tackle per hour:	
				Grade: 1368		Elite: 142	
				< 20: 2000		Grade: 152	
						< 20: 135	
Quarrie et al. (2007) [63]	26 matches		Number of match activities	1995:	2004:	NR	
		Scrums		33 ± 7	26 ± 7		
		Rucks		72 ± 18	178 ± 27		
		Mauls		33 ± 8	22 ± 9		
		Tackles		160 ± 32	270 ± 25		
Quarrie et al. (2008) [64]	434 matches	Tackle events	Total analysed	140,269		NR	
			Per game	203 ± 29			
Quarrie et al. (2012) [65]	27 matches	Scrums	Per match	Prop: 25 ± 7.8		NR	
				Hooker: 25 ± 7.6			
				Lock: 25 ± 7.9			
				Flankers: 25 ± 7.9			
				Number 8: 25 ± 7.5			
		Mauls	Per match	Prop: 1.4 ± 1.5			
				Hooker: 2 ± 2.04			
				Lock: 1.9 ± 1.9			
				Flankers: 1.8 ± 1			
				Number 8: 1.8 ± 1.4			
				Scrum Half: 0.2 ± 1			
				Fly Half: 0.2 ± 0.8			
				Midfield back: 0.3 ± 0.8			
				Wing: 0.2 ± 1			
				Full back: 0.3 ± 0.8			
		Successful tackles	Per match	Prop: 7.9 ± 3.6			
				Hooker: 9.7 ± 3.8			
				Lock: 11 ± 3.8			
				Flankers: 14 ± 4.1			
				Number 8: 12 ± 4			
				Scrum Half: 8.2 ± 3.3			
				Fly Half: 9.7 ± 3.5			
				Midfield back: 10 ± 4			
				Wing: 5.5 ± 2.7			
				Full back: 4.1 ± 2.3			
		Number of times tackled	Per match	Prop: 3.6 ± 2.6			
				Hooker: 6.2 ± 3.2			
				Lock: 4.7 ± 2.8			
				Flankers: 6.1 ± 3.4			
				Number 8: 9.7 ± 3.9			
				Scrum Half: 4.3 ± 2.7			
				Fly Half: 3.9 ± 2.6			
				Midfield back: 6.5 ± 3.1			
				Wing: 5.4 ± 2.9			
				Full back: 6.1 ± 3.1			
Reardon et al. (2017) [24]	13 matches	Collisions	Total	Prop: 33 ± 8		NR	
				Hooker: 29 ± 8			
				Second row: 33 ± 7			
				Back row: 42 ± 8			
				Scrum half: 10 ± 6			
				Out half: 19 ± 3			
				Centre: 23 ± 7			
				Wing: 22 ± 3			
				Fullback: 20 ± 5			
Reardon et al. (2017) [66]	17 matches	Collisions	NR	NR		Tight five forwards: 0.7 ± 0.6–0.8	
						Back row forwards: 0.9 ± 0.8–1.01	
						Inside backs: 0.3 ± 0.2–0.4	
						Outside backs: 0.4 ± 0.3–0.6	
Roberts et al. (2008) [68]	NR			Forwards:	Backs:	NR	
		Rucks	Number	35 ± 8	11 ± 6		
		Mauls		25 ± 8	4 ± 4		
		Scrum		21 ± 12			
		Tackle		14 ± 4	10 ± 4		
Roberts et al. (2014) [69]	30 matches (10 from each group: A, B, C)	Collisions	Total analysed	370		NR	
		Scrums	Per match	32.2			
		Tackles	Per match	140.9			
		Rucks	Per match	115.0			
		Mauls	Per match	23.4			
Schoeman et al. (2015) [73]	30 matches	Tackles	Per position	60		NR	
			Total tackles in 30 games:	Loose-head prop: 568			
				Hooker: 475			
				Tight-head prop: 553			
				Loose-head lock: 666			
				Tight-head lock: 674			
				Blind-side flank: 742			
				Open-side flank: 868			
				Eighthman: 797			
				Scrum-half: 423			
				Fly-half: 505			
				Left wing: 277			
				Inside centre: 668			
				Outside centre: 515			
				Right wing: 319			
				Full-back: 301			
			Mean collision rate/80 min:	Loose-head prop: 39.3			
				Hooker: 38.5			
				Tight-head prop: 42.1			
				Loose-head lock: 44.8			
				Tight-head lock: 41.2			
				Blind-side flank: 46.1			
				Open-side flank: 50.9			
				Eighthman: 43.1			
				Scrum-half: 16.3			
				Fly-half: 19.5			
				Left wing: 19.4			
				Inside centre: 32.3			
				Outside centre: 25.7			
				Right wing: 19.9			
				Full-back: 20.5			
			Mean tackle rate/80 min:	Loose-head prop: 12.1			
				Hooker: 11.1			
				Tight-head prop: 13.2			
				Loose-head lock: 13.7			
				Tight-head lock: 14.1			
				Blind-side flank: 16.6			
				Open-side flank: 17.3			
				Eighthman: 14.7			
				Scrum-half: 8.9			
				Fly-half: 9.4			
				Left wing: 5.2			
				Inside centre: 12.9			
				Outside centre: 9.9			
				Right wing: 6.3			
				Full-back: 5.4			
Smart et al. (2008) [74]	5 matches			Forwards:	Backs:	Forwards:	Backs:
		Tackles made	Per match	13.6 ± 7.5	6.5 ± 4.7	0.6 ± 0.2	0.2 ± 0.1
		Scrum	Number	12 ± 4.4	0		
		Scrum	Total	147.4 ± 89.8	0		
		Impact	Per match	43.6 ± 18.3	13.5 ± 7.4		
		Collisions					
Smart et al. (2014) [75]	296 matches	Tackles	Successful tackles (%)	Forwards:	Backs:	NR	
				88 ± 14	80 ± 20		
Takarada (2003) [79]	2 matches	Tackle	Mean tackles per match	14 ± 7.4		NR	
Tucker et al. (2017) [85]	1516 matches	Rucks	Per match	162.9		NR	
		Mauls	Per match	10.4			
		Tackles	Per match	158			
			Tackles/player/match	Fly half: 5			
				Scrum half: 3.8			
				Centre: 5.8			
				Full back: 2.1			
				Wing: 2.7			
				Hooker: 6.9			
				Number 8: 6.4			
				Prop: 5.5			
				Lock: 6.1			
				Flanker: 7.4			
Van Rooyen et al. (2008) [86]	7 matches	Impact contacts	Average per game	Total: 386		NR	
				Forwards: 257			
				Backs: 125			
			Scrum:	Forwards: 81			
			Ruck:	Forwards: 48			
				Backs: 8			
			Maul:	Forwards: 14			
				Backs: 4.5			
Van Rooyen et al. (2012) [87]	69 matches	Tackles	Total per match	21,886 (average 159 ± 42)		NR	
			6 Nations	165 ± 28			
			Tri Nations	141 ± 24			
			RWC	156 ± 47			
Van Rooyen et al. (2014) [88]	15 matches	Tackle	Tackle situations per match	Average: 191 ± 32		NR	
				Average winning team: 89 ± 30			
				Average losing team: 101 ± 24			
Vaz et al. (2010) [89]	ons: 64 matches	Tackles made:	Total	Winners:	Losers:	NR	
				88 ± 27.6	89 ± 37.8		
	S12 competition: 95 matches			112.7 ± 33.1	99.4 ± 30		
Vaz et al. (2012) [90]	Training session (Small sided games)	Tackles	Tackles made:	Novice:	Experienced:	NR	
				28.2 ± 3.3	48.7 ± 3.3		
Villarejo et al. (2013) [92]	48 matches	Tackles	Attempted tackles	Front row: 10		NR	
				Second row: 10.9			
				Back row: 14.3			
				Scrum halves: 12.5			
				Middle backs: 10.5			
				Back three: 5.9			
			Tackles made	Front row: 8			
				Second row: 8.6			
				Back row: 11.2			
				Scrum halves: 8.3			
				Middle backs: 7.2			
				Back three: 3.7			
			Ineffective tackles	Front row: 0.7			
				Second row: 0.6			
				Back row: 1.1			
				Scrum halves: 1.7			
				Middle backs: 1.2			
				Back three: 0.9			
Villarejo et al. (2015) [93]	48 matches	Tackles	Attempted tackles	Winning team:	Losing team:	NR	
				Front row: 10.5 ± 14.04	Front row: 9.4 ± 12.4		
				Second row: 10.2 ± 8.6	Second row: 11.6 ± 14.9		
				Back row: 14.5 ± 14.6	Back row: 14.2 ± 17.6		
				Scrum halves: 9.5 ± 11.1	Scrum halves: 15.3 ± 24.7		
				Inside backs: 9.3 ± 12.9	Inside backs: 11.4 ± 10.6		
				Outside backs: 5.5 ± 9.6	Outside backs: 6.2 ± 7.4		
			Effective tackles:	Front row: 8.9 ± 12.9	Front row: 6.8 ± 9.8		
				Second row: 8.4 ± 7.3	Second row: 8.7 ± 9.5		
				Back row: 12 ± 11.6	Back row: 10.6 ± 14.9		
				Scrum halves: 7.5 ± 9.3	Scrum halves: 8.8 ± 15.4		
				Inside backs: 7.02 ± 10.9	Inside backs: 7.1 ± 7.2		
				Outside backs: 4 ± 7.5	Outside backs: 3.3 ± 3.7		
			Ineffective tackles:	Front row: 0.5 ± 2	Front row: 0.9 ± 2.4		
				Second row: 0.5 ± 1.1	Second row: 0.8 ± 1.5		
				Back row: 1 ± 4.1	Back row: 1.1 ± 2.8		
				Scrum halves: 1.1 ± 3.1	Scrum halves: 2.3 ± 6		
				Inside backs: 0.7 ± 2.03	Inside backs: 1.5 ± 2.8		
				Outside backs: 0.5 ± 1.7	Outside backs: 1.4 ± 6.1		
Virr et al. (2014) [94]	10 matches	Ruck/maul/tackle	Total number	Forwards:	Backs:	NR	
		Scrum		61 ± 12	25 ± 11		
				33 ± 7			
*Rugby sevens*							
Clarke et al. (2016) [37]	2 matches	Collisions	Collisions	Men: 51		NR	
				Women: 44			
Hendricks et al. (2019) [3]	135 matches	Tackles	Per match	1.9 ± 1.3		NR	
			Total	8.4 ± 4.1			
		Ruck	Total	0.4 ± 0.7			
Higham et al. (2014) [5]	196 matches	Scrums	Per team per match			NR	
		Rucks	Per team per match				
		Mauls	Per team per match				
Peeters et al. (2019) [60]	32 matches	Contact actions	Tackles/collisions/rucks/ mauls	Forwards:	Backs:	NR	
				First half: 5.3 ± 2.8	First half: 5.3 ± 3		
				Second half: 6.3 ± 2.9	Second half: 6.1 ± 2.7		
Reyneke et al. (2018) [67]	15 matches	Tackles:	Low (< 21 score):	3.4 ± 1.8		NR	
			High (>/ = 21 score):	3 ± 2			
		Scrums	Low (< 21 score):	1.6 ± 1.3			
			High (>/ = 21 score):	1.2 ± 1.8			
		Ball Carry	Low (< 21 score):	4.4 ± 2.9			
			High (>/ = 21 score):	4.9 ± 2.5			
Ross et al. (2015) [70]	NR	Tackles:	Total	NR			
			Provincial:			0.2 ± 0.1	
			International:			0.2 ± 0.2	
		Rucks:	Provincial:			0.1 ± 0.1	
			International:			0.2 ± 0.2	
		Ball Carries:	Provincial:			0.3 ± 0.2	
			International:			0.2 ± 0.2	
Ross et al. (2015) [71]	54 matches			Forwards:	Backs:	NR	
		Tackles	Per match	2.7 ± 2.6	2.41 ± 2.5		
		Scrums		1.8 ± 1.9			
		Ball Carries		3.2 ± 2.4	4.1 ± 3.2		
Ross et al. (2016) [72]	37 matches (between team analysis)	Tackles	Dominant tackles per match:	2.1 ± 2.3		NR	
	50 matches (single team analysis)		Ineffective tackles:	8.1 ± 3.9			
		Rucks	Defensive ruck average per match:	1.2 ± 0.3			
			Ruck average:	1.2 ± 0.2

5. Fig. 5c: The two entries for Vaz et al. 2010 (89) have now been removed from this figure. The original version of Fig. 5 has been replaced with the version shown below:

**Fig. 5:**
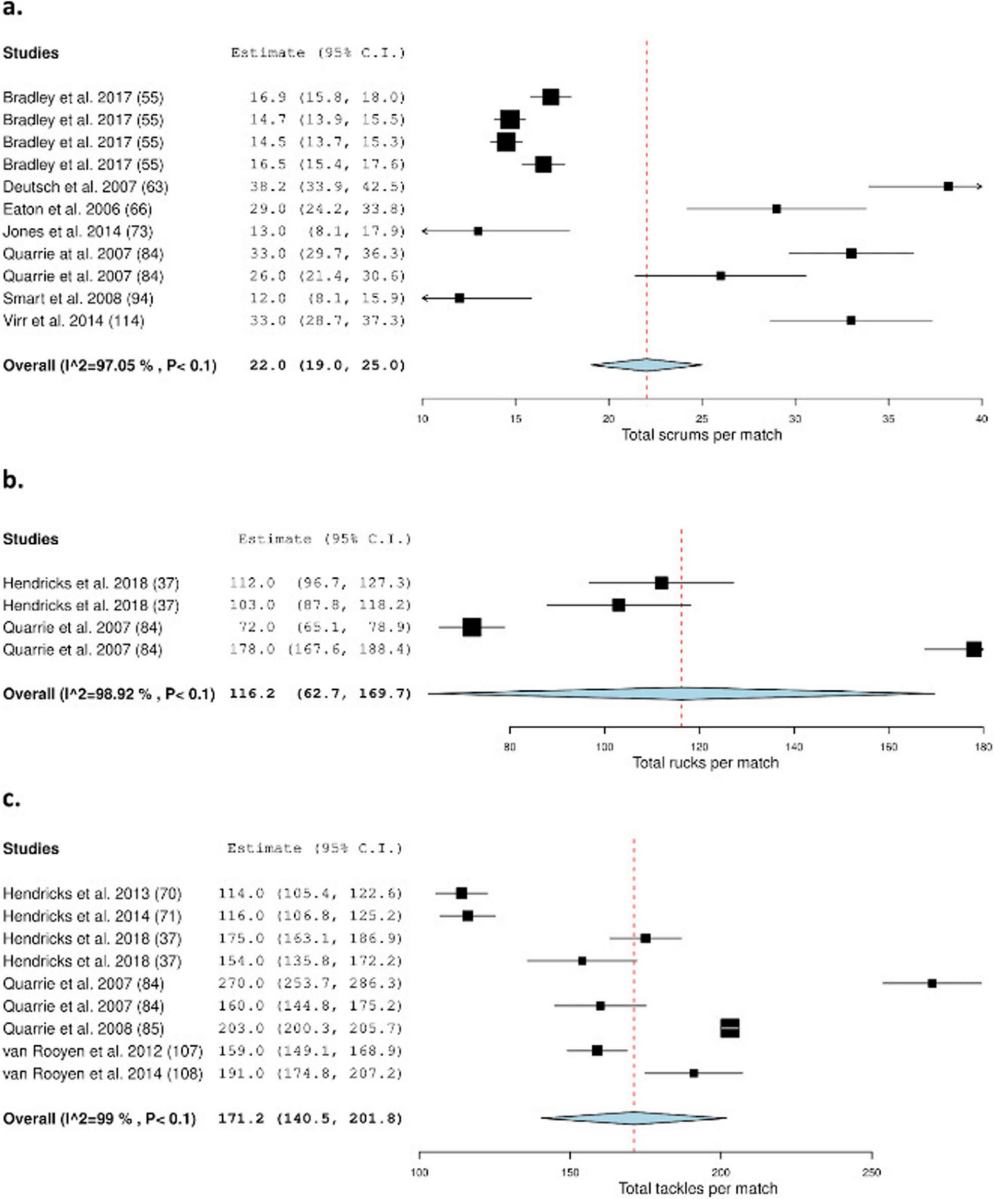
Meta-analysis of studies reporting absolute total scrums, rucks, and tackles per match (n) from video-based analysis in rugby union. The forest plot (mean and 95% confidence interval (CI)) presents the results of the meta-analysis of the pooled data estimates for the total **a** scrums, **b** rucks and **c** tackles per match. The squares and horizontal lines represent individual study mean and 95% CI and the diamond presents the pooled mean and 95% CI. The bigger the square the larger the sample size

