# Supplementary material for: Quantifying Collision Frequency and Intensity in Rugby Union and Rugby Sevens: A Systematic Review
Source: Sports Med Open. 2022 Jan 20;8:12. doi: 10.1186/s40798-021-00398-4 (PMC8776953; doi:10.1186/s40798-021-00398-4)
Supplement: Supplementary file 1 — Additional file 1: Table S1. Methodological quality assessment of the final full text articles according to Downs et al. [30]. Table S2. Characteristics of studies using microtechnology to record collisions during match-play or training sessions. Table S3. Characteristics of studies using video-based analysis to record collisions during match-play or training sessions. [file 40798_2021_398_MOESM1_ESM.docx]

**Table S1**. Methodological quality assessment of the final full text articles according to Downs et al. (1998) (30).

| Study: Author (year) |  | Question number | | | | | | | | | | | Total Score |
| --- | --- | --- | --- | --- | --- | --- | --- | --- | --- | --- | --- | --- | --- |
|  | **1** | **2** | **3** | **4** | **6** | **7** | **10** | **11** | **12** | **16** | **18** | **20** |  |
| Austin et al. (2011) (31) | 1 | 1 | 1 | 1 | 1 | 1 | 0 | 1 | UT | 1 | 1 | 1 | 10 |
| Bradley et al.  (2015) (32) | 1 | 1 | 1 | 1 | 1 | 1 | 1 | 0 | UT | 1 | 1 | 1 | 10 |
| Bradley et al.  (2017) (33) | 1 | 1 | 0 | 1 | 1 | 1 | 1 | 1 | UT | 1 | 1 | 1 | 10 |
| Campbell et al. (2017) (34) | 1 | 1 | 0 | 1 | 1 | 1 | 1 | 1 | UT | 1 | 1 | 1 | 10 |
| Clarke et al.  (2015) (35) | 1 | 1 | 1 | 1 | 1 | 1 | 1 | 1 | UT | 1 | 1 | 1 | 11 |
| Clarke et al.  (2015) (36) | 1 | 1 | 1 | 1 | 1 | 1 | 1 | 1 | UT | 1 | 1 | 1 | 11 |
| Clarke et al.  (2016) (37) | 1 | 1 | 1 | 1 | 1 | 1 | 0 | UT | UT | 1 | 1 | 1 | 8 |
| Clarke et al.  (2017) (38) | 1 | 1 | 1 | 1 | 1 | 1 | 1 | 1 | UT | 1 | 1 | 1 | 11 |
| Coughlan et al.  (2011) (39) | 1 | 1 | 1 | 1 | 1 | 0 | 0 | 0 | UT | 1 | 0 | 1 | 7 |
| Cunniffe et al. (2009) (20) | 1 | 1 | 1 | 1 | 1 | 0 | 0 | 0 | UT | 1 | 0 | 0 | 6 |
| Deutsch et al.  (1998) (40) | 1 | 1 | 1 | 1 | 1 | 1 | 0 | 1 | UT | 1 | 1 | 1 | 10 |
| Deutsch et al.  (2007) (41) | 1 | 1 | 0 | 1 | 1 | 1 | 0 | 1 | UT | 1 | 1 | 1 | 9 |
| Dubois et al. (2020) (42) | 1 | 1 | 1 | 1 | 1 | 1 | 1 | 1 | UT | 1 | 1 | 1 | 11 |
| Duthie et al.  (2005) (43) | 1 | 1 | 0 | 1 | 1 | 1 | 0 | 1 | UT | 1 | 1 | 1 | 9 |
| Eaton et al. (2006) (44) | 1 | 1 | 0 | 1 | 1 | 1 | 1 | 1 | UT | 1 | 1 | 1 | 10 |
| Fuller et al.  (2007) (45) | 1 | 1 | 0 | 1 | 1 | 1 | 1 | 1 | UT | 1 | 1 | 1 | 10 |
| Fuller et al.  (2008) (46) | 1 | 1 | 0 | 1 | 1 | 1 | 1 | 1 | UT | 0 | 1 | 1 | 9 |
| Gibson et al. (2015) (47) | 1 | 1 | 1 | 1 | 1 | 1 | 1 | UT | UT | 1 | 1 | 1 | 10 |
| Grainger et al.  (2018) (48) | 1 | 1 | 1 | 1 | 1 | 1 | 1 | 1 | UT | 1 | 1 | 1 | 11 |
| Hendricks et al. (2013) (49) | 1 | 1 | 0 | 1 | 1 | 1 | 1 | 1 | UT | 1 | 1 | 1 | 10 |
| Hendricks et al. (2014) (50) | 1 | 1 | 0 | 1 | 1 | 1 | 0 | 1 | UT | 1 | 1 | 1 | 9 |
| Hendricks et al. (2018) (8) | 1 | 1 | 0 | 1 | 1 | 1 | 1 | 1 | UT | 1 | 1 | 1 | 10 |
| Hendricks et al. (2019) (3) | 1 | 1 | 0 | 1 | 1 | 1 | 0 | 1 | UT | 1 | 1 | 1 | 9 |
| Higham et al. (2014) (5) | 1 | 1 | 1 | 1 | 1 | 1 | 0 | 1 | UT | 1 | 1 | 1 | 10 |
| Higham et al. (2016) (51) | 1 | 1 | 1 | 1 | 1 | 1 | 0 | 1 | UT | 1 | 1 | 1 | 9 |
| Jones et al. (2014) (52) | 1 | 1 | 1 | 1 | 1 | 1 | 0 | 1 | UT | 1 | 1 | 1 | 10 |
| Jones et al. (2015)  (53) | 1 | 1 | 0 | 1 | 1 | 1 | 0 | 1 | UT | 1 | 1 | 1 | 9 |
| Lacome et al.  (2016) (54) | 0 | 1 | 0 | 1 | 1 | 1 | 1 | 1 | UT | 1 | 1 | 1 | 9 |
| Lindsay et al.  (2015) (55) | 1 | 1 | 1 | 1 | 1 | 1 | 0 | 1 | UT | 1 | 1 | 1 | 10 |
| Lindsay et al.  (2017) (56) | 1 | 1 | 1 | 1 | 1 | 1 | 1 | 1 | UT | 1 | 1 | 1 | 11 |
| MacLeod et al.  (2018) (25) | 1 | 1 | 1 | 1 | 1 | 1 | 1 | 1 | UT | 1 | 1 | 1 | 11 |
| McIntosh et al.  (2010) (57) | 0 | 1 | 0 | 1 | 1 | 1 | 1 | 1 | UT | 1 | 1 | 1 | 9 |
| McLaren et al.  (2015) (58) | 1 | 1 | 1 | 1 | 1 | 1 | 0 | 1 | UT | 1 | 0 | 1 | 9 |
| McLellan et al. (2013) (29) | 1 | 1 | 1 | 1 | 1 | 1 | 0 | 0 | UT | 1 | 1 | 1 | 9 |
| Owen et al.  (2015) (59) | 1 | 1 | 1 | 1 | 1 | 1 | 1 | 1 | UT | 1 | 1 | 1 | 11 |
| Peeters et al.  (2019) (60) | 1 | 1 | 1 | 1 | 1 | 1 | 1 | 1 | UT | 1 | 0 | 1 | 10 |
| Pollard et al.  (2018) (61) | 1 | 1 | 1 | 1 | 1 | 1 | 0 | 1 | UT | 1 | 1 | 1 | 10 |
| Portillo et al.  (2016) (62) | 1 | 1 | 1 | 1 | 1 | 1 | 1 | 1 | UT | 1 | 1 | 1 | 11 |
| Quarrie et al. (2007) (63) | 1 | 1 | 0 | 1 | 1 | 1 | 0 | 1 | UT | 1 | 0 | 0 | 7 |
| Quarrie et al.  (2008) (64) | 1 | 1 | 0 | 1 | 1 | 1 | 0 | 1 | UT | 1 | 1 | 1 | 9 |
| Quarrie et al.  (2012) (65) | 1 | 1 | 0 | 1 | 1 | 1 | 0 | 1 | UT | 1 | 1 | 1 | 9 |
| Reardon et al.  (2017) (24) | 1 | 1 | 1 | 1 | 1 | 1 | 1 | 1 | UT | 1 | 1 | 1 | 11 |
| Reardon et al.  (2017) (66) | 1 | 1 | 1 | 1 | 1 | 1 | 0 | 1 | UT | 1 | 1 | 1 | 10 |
| Reyneke et al.  (2018) (67) | 1 | 1 | 1 | 1 | 1 | 1 | 0 | 1 | UT | 1 | 0 | 1 | 9 |
| Roberts et al.  (2008) (68) | 1 | 1 | 0 | 1 | 1 | 1 | 1 | 1 | UT | 1 | 1 | 1 | 10 |
| Roberts et al.  (2014) (69) | 1 | 1 | 0 | 1 | 1 | 1 | 0 | 1 | UT | 1 | 1 | 1 | 10 |
| Ross et al.  (2015) (70) | 1 | 1 | 0 | 1 | 1 | 1 | 1 | 1 | UT | 1 | 1 | 1 | 10 |
| Ross et al. (2015)  (71) | 1 | 1 | 1 | 1 | 1 | 1 | 1 | 1 | UT | 1 | 1 | 1 | 11 |
| Ross et al. (2016) (72) | 1 | 1 | 0 | 1 | 1 | 1 | 0 | 1 | UT | 1 | 1 | 1 | 9 |
| Schoeman et al.  (2015) (73) | 1 | 1 | 0 | 1 | 1 | 1 | 1 | 1 | UT | 1 | 1 | 1 | 10 |
| Smart et al. (2008) (74) | 1 | 1 | 1 | 1 | 1 | 1 | 0 | 1 | UT | 1 | 1 | 1 | 10 |
| Smart et al.  (2014) (75) | 1 | 1 | 0 | 1 | 1 | 1 | 1 | 1 | UT | 1 | 1 | 1 | 10 |
| Suarez-Arrones et al. (2012) (76) | 1 | 1 | 1 | 1 | 1 | 1 | 0 | 1 | UT | 1 | 1 | 1 | 10 |
| Suarez-Arrones et al. (2013) (77) | 1 | 1 | 1 | 1 | 1 | 1 | 1 | 1 | UT | 1 | 1 | 1 | 10 |
| Suarez-Arrones et al. (2014) (78) | 1 | 1 | 1 | 1 | 1 | 1 | 1 | 1 | UT | 1 | 1 | 1 | 11 |
| Takarada (2003) (79) | 1 | 1 | 1 | 1 | 1 | 1 | 0 | 1 | UT | 1 | 1 | 1 | 10 |
| Takeda et al. (2014) (80) | 1 | 1 | 1 | 1 | 1 | 1 | 0 | 1 | UT | 1 | 1 | 0 | 9 |
| Tee et al. (2015) (81) | 1 | 1 | 1 | 1 | 1 | 1 | 1 | 1 | UT | 1 | 1 | 1 | 11 |
| Tee et al. (2017) (82) | 1 | 1 | 1 | 1 | 1 | 1 | 0 | 1 | UT | 1 | 0 | 1 | 9 |
| Tee et al. (2020) (83) | 1 | 1 | 1 | 1 | 1 | 1 | 1 | 1 | UT | 1 | 1 | 1 | 11 |
| Tierney et al. (2020) (23) | 1 | 1 | 1 | 1 | 1 | 1 | 1 | 1 | UT | 1 | 1 | 1 | 11 |
| Tierney et al. (2021) (84) | 1 | 1 | 1 | 1 | 1 | 1 | 1 | 1 | UT | 1 | 1 | 1 | 11 |
| Tucker et al.  (2017) (85) | 1 | 1 | 0 | 1 | 1 | 1 | 0 | 1 | UT | 1 | 1 | 1 | 9 |
| Van Rooyen et al.  (2008) (86) | 1 | 1 | 1 | 1 | 1 | 1 | 0 | 1 | UT | 1 | 0 | 1 | 9 |
| Van Rooyen et al.  (2012) (87) | 1 | 1 | 0 | 1 | 1 | 1 | 0 | 1 | UT | 1 | 1 | 1 | 9 |
| Van Rooyen et al.  (2014) (88) | 1 | 1 | 0 | 1 | 1 | 1 | 1 | 1 | UT | 1 | 1 | 1 | 10 |
| Vaz et al. (2010) (89) | 1 | 1 | 0 | 1 | 1 | 1 | 0 | 1 | UT | 1 | 1 | 1 | 9 |
| Vaz et al. (2012) (90) | 1 | 1 | 1 | 1 | 1 | 1 | 1 | 1 | UT | 1 | 1 | 1 | 11 |
| Venter et al.  (2011) (91) | 1 | 1 | 1 | 1 | 1 | 1 | 0 | 1 | UT | 1 | 1 | 1 | 10 |
| Villarejo et al.  (2013) (92) | 1 | 1 | 0 | 1 | 1 | 0 | 0 | 1 | UT | 1 | 1 | 1 | 8 |
| Villarejo et al.  (2015) (93) | 1 | 1 | 0 | 1 | 1 | 1 | 1 | 1 | UT | 1 | 1 | 1 | 10 |
| Virr et al.  (2014) (94) | 1 | 1 | 1 | 1 | 1 | 1 | 0 | 1 | UT | 1 | 1 | 1 | 10 |
| Yamamoto et al. (2020) (95) | 1 | 1 | 1 | 1 | 1 | 1 | 0 | 1 | UT | 1 | 1 | 1 | 10 |

1. Is the hypothesis/aim/objectives of the study clearly described? 2. Are the main outcomes to be measured clearly described in the introduction or methods section? 3. Are the characteristics of the participants included in the study clearly described? 6. Are the main findings of the study clearly described? 7. Does the study provide estimates of the variability in the data for the main outcomes? 10. Have the actual probability values/effect sizes been reported (e.g.0.035 rather than <0.05) for the main outcome except where the probability value is less than 0.001? 11. Were the subjects asked to participate in the study representative of the entire population from which they were recruited? 12. Were the subjects who were prepared to participate representative of the entire population from which they were recruited? 16. If any of the results of the study were based on ‘data dredging’, was this made clear? 18. Were the statistical tests used to assess the main outcomes appropriate? 20. Were the main outcome measures used accurate (valid and reliable)? UT: Unable to determine

**Table S2.** Characteristics of studies using microtechnology to record collisions during match-play or training sessions.

| Study: Author (year) | GPS provider: | Device (algorithm) | GPS device rate (Hz): | Accelerometer (100Hz): | Gyroscope: | Number of files | Software: |
| --- | --- | --- | --- | --- | --- | --- | --- |
| Bradley et al. (2015) (32) | Catapult Innovations, Melbourne, Australia | Minimax S4 | 10 | Yes | Yes | NR | NR |
| Campbell et al. (2017) (34) | GPSports Systems, Canberra, Australia | SPI HPU | 15 | NR | NR | NR | Team AMS, GPSports, Canberra, Australia |
| Clarke et al.  (2015) (35) | GPSports Systems, Canberra, Australia | SPI HPU | 5 | NR | NR | 64: National, 51 State | NR |
| Clarke et al. (2015) (36) | GPSports Systems, Canberra, Australia | SPI HPU | 15 | Yes | NR | 64: National, 51 State | NR |
|  |  |  |  |  |  |  |  |
| Clarke et al.  (2016) (37) | GPSports Systems, Canberra, Australia | SPI HPU | NR | Yes | NR | NR | NR |
| Clarke et al.  (2017) (38) | GPSports Systems, Canberra, Australia | SPI HPU | 5 | NR | NR | Senior men:68  Senior women: 90  Elite men: 81  Elite women: 89 | NR |
| Coughlan et al.  (2011) (39) | GPSports Systems, Canberra, Australia | SPI-Pro | 5 | Yes | Yes | NR | Team AMS ; GPSports, Version 1.2, Canberra, Australia |
| Cunniffe et al. (2009) (20) | GPSports  Systems, Canberra, Australian Capital Territory, Australia | SPI Elite | 1 | Yes | NR | NR | Team AMS ; GPSports, Version 1.2, Canberra, Australia |
| Dubois et al. (2020) (42) | GPSports Systems, Canberra, Australia | SPI HPU | 5 | Yes | NR | NR | NR |
| Gibson et al. (2015) (47) | Catapult Innovations, Melbourne, Australia | Minimax S4 | 10 | Yes | Yes | 136 | Sprint 5.14 software |
| Grainger et al.  (2018) (48) | StatSports Viper, Northern Ireland | NR | 10 | Yes | NR | 462 | STATSports Viper Rugby software |
| Higham et al. (2016) (51) | GPSports Systems, Canberra, Australia | SPI Pro X | 15 | Yes | NR | NR | Team AMS release 1 2011 revision 8, GPSports Systems |
| Jones et al. (2014) (52) | Catapult Innovations, Melbourne, Australia | Minimax S4, v.4.0 | 10 | NR | NR | 45 | Catapult Sprint software (Catapult Innovations, Melbourne, Australia) |
| Jones et al. (2015) (53) | Catapult Innovations, Melbourne, Australia | Minimax S4, v.4.0 | 100 | Yes | NR | 112 | Catapult Sprint software (Catapult Innovations, Melbourne, Australia) |
| Lindsay et al.  (2017) (56) | Catapult, Victoria, Australia | Minimax S4 | 10 | Yes | NR | NR | Catapult Sprint V5.1 |
| MacLeod et al.  (2018) (25) | STATSport Viper unit | NR | 10 | Yes | NR | NR | STATSports Viper Rugby software |
| McLaren et al.  (2015) (58) | Catapult Innovations, Melbourne, Australia | Minimax S4 | 10 | Yes | Yes | NR | Logan Plus 4.2 software (Catapult Innovations, Melbourne, Australia) |
| McLellan et al. (2013) (29) | GPSports Systems, Canberra, Australian Capital Territory, Australia | SPI-Pro | 15 | Yes | NR | NR | SPi-Ezy v2.1 software (GPSports Systems). |
| Owen et al.  (2015) (59) | GPSports Systems, Canberra, Australia | SPI HPU | 15 | Yes | NR | 189 | Team AMS ; GPSports, Canberra, Australia |
| Pollard et al.  (2018) (61) | STATSports, Belfast, UK | Viper Pod | 10 | NR | NR | 4.5 (2.6) per player | Viper PSA Software, Version 2.6.1.176, STATSports, Belfast, UK |
| Portillo et al.  (2016) (62) | GPSports, Canberra, Australia | NR | 100 | Yes | NR | NR | Team AMS software V R1.2011.6, GPS sports, Canberra, Australia |
| Reardon et al.  (2017) (24) | Catapult, Innovations, Scoresby, VIC, Australia | OptimEyeS5 | 10 | Yes | Yes | 135 | Sprint 5.1 software (Catapult Innovations, Scoresby, VIC, Australia) |
| Reardon et al.  (2017) (66) | Catapult, Melbourne, Australia | OptimEyeS5 | 10 | NR | NR | 200 | Sprint 5.1 software (Catapult Innovations, Scoresby, VIC, Australia) |
| Suarez-Arrones et al. (2012) (76) | GPsports, Australia | SPI Elite | 1 | Yes | NR | NR | Team AMS ; GPSports, Version 1.2, Canberra, Australia |
| Suarez-Arrones et al. (2013) (77) | GPSports Systems, Canberra, Australia | SPI Pro X | 5 | Yes | NR | NR | Team AMS software version 10 (GPSports, Australia) |
| Suarez-Arrones et al. (2014) (78) | GPSports Systems, Canberra, Australia | SPI Pro X | 15 | Yes | NR | NR | NR |
| Tee et al. (2015) (81) | GPSports, Australia | SPI Pro | 5 | Yes | NR | 102 | Team AMS software version 10 (GPSports, Australia) |
| Tee et al. (2017) (82) | GPSports, Canberra, Australia | SPI Pro | 5 | Yes | NR | 46 | Team AMS software (version 10) |
| Tee et al. (2020)  (83) | GPSports, Canberra, Australia | SPI Pro | 10 | Yes | NR | 103 | Team AMS software version 10 (GPSports, Australia) |
| Tierney et al. (2020) (23) | StatSports Group Limited, Co. Down, Northern Ireland | MST unit (Weighted algorithm) | 10 | Yes (600Hz) | Yes | 245 | StatSports Rugby software |
| Tierney et al. (2021) (84) | StatSports, Down, UK | Apex Pro Series Pod | 10 | Yes (600Hz) | Yes | 2869 | StatSports Apex Rugby Software (Version 3.0.11171) |
| Vaz et al. (2012) (90) | GPSports Systems, Canberra, Australia | SPI Pro | 5 | NR | NR | NR | Team AMS, V2.1.0.5 P11 software |
| Venter et al.  (2011) (91) | GPSports Systems, Canberra, Australia | SPI Pro | 5 | NR | NR | 23 | Team AMS ; GPSports, Canberra, Australia |
| Yamamoto et al. (2020) (95) | GPSports Systems,  Canberra, Australia | SPI Pro X | 5 | Yes | NR | NR | Team AMS; GPSports Systems |

NR= Not reported

**Table S3.** Characteristics of studies using video-based analysis to record collisions during match-play or training sessions.

| Study: Author (year) | Camera system | Number of cameras | Location of camera relative to field | Notational analysis system |
| --- | --- | --- | --- | --- |
| Austin et al. (2011) (31) | HITACHI DZ-GX5060SW, Hitachi LTD., Japan | 3 | On the halfway line approximately 30m above the playing field | A simple hand-notation game analysis system |
| Bradley et al. (2017) (33) | VirginMedia TiVo box | NR | NR | NR |
| Campbell et al. (2017) (34) | Legria HF R506, Canon, Tokyo, Japan | NR | 3-5 m above the height of the playing field. The footage was taken from a vantage point 10-20 m from the field either side of the 22 m and halfway lines. | NR |
| Coughlan et al. (2011) (39) | NR | NR | NR | Sportscode Pro, Version 7.5.6, Sportstec, Warriewood, NSW, Australia |
| Deutsch et al. (1998) (40) | Panasonic MS-4, Matsushita Electronics, Japan | 3 | 5± 6 m away from the side line at half-way, at an elevation of 3± 6 m. | NR |
| Deutsch et al. (2007) (41) | Panasonic MS-4, Matsushita Electronics, Japan | NR | 5 and 15 m from the field, at an elevation of 5 – 10 m | Pro-Log, Time Frame International, New Zealand |
| Duthie et al. (2005) (43) | SONY DCR-TRV900E PAL, Sony Corporation of America | 1 | 20 m above the ground midpoint of the rugby field | Part-Timer V1.1, Australian Sports Commission, Canberra |
| Eaton et al. (2006) (44) | NR | 3 | Image recognition sensors were fixed to the roof of the Northampton Saint’s stadium. | Prozone |
| Fuller et al. (2007) (45) | DVD recordings from Rugby Football Union | NR | NR | NR |
| Fuller et al. (2008) (46) | DVD recordings from Rugby Football Union | NR | NR | NR |
| Hendricks et al. (2013) (49) | NR | NR | NR | Sports Code Elite Version 6.5.1, using an Apple iMac (Apple, USA) |
| Hendricks et al. (2014) (50) | NR | NR | NR | Sports Code elite version 6.5.1, using an Apple iMac (Apple, USA) |
| Hendricks et al. (2018) (8) | NR | NR | NR | Sports Code elite version 6.5.1, using an Apple iMac (Apple, USA) |
| Hendricks et al. (2019) (3) | NR | NR | NR | Sports Code elite version 6.5.1, using an Apple iMac (Apple, USA) |
| Jones et al. (2014) (52) | NR | NR | NR | Sportscode (Sportstec, NSW) |
| Lacome et al. (2016) (54) | Amisco, Pro, Sport Universal Process, Nice, France | NR | NR | NR |
| Lindsay et al. (2015) (55) | NR | NR | NR | Opta provided live performance data analysis |
| Lindsay et al. (2017) (56) | NR | NR | NR | Opta provided live performance data analysis |
| Macleod et al. (2018) (25) | NR | NR | NR | Sportscode, Version 8.4.0, Sportstec, NSW, Australia |
| McIntosh et al.  (2010) (57) | NR | NR | NR | Snapperi (Webbsoft Technologies, Australia) |
| Peeters et al. (2019) (60) | NR | NR | NR | SportsCode, Hudl, USA |
| Quarrie et al. (2007) (63) | NR | NR | NR | Verusco Ltd., Palmerston North; [www.verusco.com](http://www.verusco.com) |
| Quarrie et al. (2008) (64) | NR | NR | NR | Verusco Technologies, Palmerston North, New Zealand |
| Quarrie et al. (2012) (65) | NR | NR | NR | Verusco Technologies Inc. (Palmerston North, New Zealand) |
| Reardon et al. (2017) (24) | NR | NR | NR | SportscodeTM (Sportstec by Hudl, Nebraska, USA) |
| Reyneke et al. (2018) (67) | NR | NR | NR | Sportscode V8.9, Sportstec, Australia |
| Roberts et al. (2008) (68) | Four Sony DCR-TRV900E, Japan; one Panasonic AG DP2000B, Japan | 5 | 3–5 m from the nearest side-line | V9 time code generator, IMP Electronics, Cambridgeshire |
| Roberts et al. (2014) (69) | Sony DCR-TRV900E, Japan | 1 | Mounted on a tripod | SportsCode Pro 7.0.150, Sportstec, Australia |
| Ross et al. (2015) (70) | NR | NR | NR | Sportscode V8.9, Sportstec, Australia |
| Ross et al. (2015) (71) | NR | NR | NR | Sportscode V8.9, Sportstec,  Australia |
| Ross et al. (2016) (72) | NR | NR | NR | Sportscode V8.9, Sportstec,  Australia |
| Schoeman et al.  (2015) (73) | Supplied by Cheetahs Super Rugby Franchise | NR | NR | Verusco TryMaker Pro (Verusco Technologies Ltd.; Palmerston North, New Zealand) |
| Smart et al. (2008) (74) | NR | NR | NR | AnalySports, Version AS10.0307, 2002, Palmerston North, NZ |
| Smart et al. (2014) (75) | NR | NR | NR | TryMaker Pro, Verusco Ltd, Palmerston North, New Zealand |
| Tierney et al. (2020) (23) | NR | NR | NR | Sportscode™ (Sportstec  by Hudl, Nebraska, USA) |
| Van Rooyen et al.  (2008) (86) | NR | NR | NR | SportsCode  Elite version 6.5.2, Sportstec, Australia |
| Van Rooyen et al.  (2012) (87) | NR | NR | NR | ‘Verusco Statistics Portal’ (Verusco Sports 2012) |
| Van Rooyen et al.  (2014) (88) | NR | NR | NR | SportsCode Elite version 6.5.1, Sportstec, Australia |
| Vaz et al. (2010) (89) | NR | NR | NR | Rugby Stats Fair Play Sports Analysis Systems  V2, Australia) and Rugby Match Analysis and Statistics  (IRB - Computacenter/S.A.S, 2003) |
| Vaz et al. (2012) (90) | Sony HDR–XR 155E-120G HandyCam; Japan |  | 20 m from the side of the field at an elevation of 3 m |  |
| Virr et al. (2014) (94) | Sony GR-DVL9800, Son Corporation, Tokoyo, Japan; Panasonic PV-GS150, Panasonic Corporation, Osaka, Japan; JVC GR-DVL520U, Victor Company of Japan, Ltd., Yokohama, Japan | 4 | Centre line (50m line) on 1.6 m tripods situated on top of a 2 m, scaffold, 5 m from the sideline | Dartfish TeamPro software, version 4.0.6.0, Dartfish, Fribourg, Switzerland |

NR= Not reported
